# The Effects of Beetroot Juice Supplementation on Performance and Fatigue During Single and Repeated Sprints: A Systematic Review and Meta-Analysis

**DOI:** 10.3390/nu18152513

**Published:** 2026-08-03

**Authors:** Melike Nur Eroglu, Serkan Pancar, Olga López-Torres, Valentín Emilio Fernández-Elías

**Affiliations:** 1 Coaching Education Department, Faculty of Sport Sciences, Sakarya University of Applied Sciences, Sakarya 54050, Turkey; melikeeroglu@subu.edu.tr; 2 Coaching Education Department, Faculty of Sport Sciences, Aksaray University, Aksaray 68100, Turkey; serkanpancar@aksaray.edu.tr; 3 Faculty of Medicine, Health and Sport, Universidad Europea de Madrid, 28670 Villaviciosa de Odón, Spain; olga.lopez@universidadeuropea.es; 4 Research Center in Sport Sciences, Universidad Rey Juan Carlos, 28942 Fuenlabrada, Spain

**Keywords:** beetroot juice, dietary nitrate, sprint performance, repeated sprint ability, fatigue, power output, neuromuscular performance

## Abstract

**Background/Objectives**: Beetroot juice (BRJ), a dietary nitrate source, may enhance high-intensity intermittent exercise, but its effects on single- and repeated-sprint performance remain unclear. This systematic review and meta-analysis aimed to evaluate the effects of BRJ supplementation on sprint-related performance and neuromuscular, perceptual, and physiological outcomes. **Methods**: PubMed, Scopus, and Web of Science were searched for trials published from 2014 to June 2026. Eligible studies compared BRJ with placebo or control in healthy adults aged 18–40 years. The review followed PRISMA guidelines. Methodological quality and risk of bias were assessed using the PEDro scale and Cochrane RoB 2 tool. Evidence certainty was assessed using GRADE. Standardized mean differences (SMDs) with 95% confidence intervals (CIs) were calculated. **Results**: Twenty-three randomized trials involving 401 participants were included, and 21 contributed to the meta-analysis. BRJ improved time to peak power in four Wingate-based studies (SMD = −0.92, 95% CI: −1.29 to −0.54, *p* < 0.001) and handgrip strength (SMD = 0.40, 95% CI: 0.05 to 0.74, *p* = 0.027). No significant effects were observed for countermovement jump, peak or mean power, 10 m or 20 m sprint performance, perceived exertion, heart rate, or blood lactate. Sex did not significantly moderate the effects, although female data were limited. Certainty was low for most outcomes and very low for 20 m sprint performance and blood lactate. **Conclusions**: BRJ may improve time to peak power and handgrip strength; however, low- to very-low-certainty evidence does not support its routine use to enhance short-distance or repeated-sprint performance.

## 1. Introduction

Team sports such as soccer, rugby, basketball and field hockey are characterized by intermittent activity patterns that require athletes to perform frequent maximal or near-maximal sprint efforts interspersed with brief recovery periods [1,2]. Sprint-based performance is therefore an important determinant of success in these sports and can be broadly categorized into single-sprint and repeated-sprint exercise. Single-sprint performance reflects the ability to generate maximal force and power over a very short duration and is influenced by neuromuscular function, phosphocreatine availability, and the rapid activation of type II muscle fibers [3]. In contrast, repeated-sprint ability (RSA) describes the capacity to perform multiple short-duration sprints, typically lasting less than 10 s, with limited recovery between efforts [1,4]. This type of exercise places high demands on fatigue tolerance, metabolic recovery, and the maintenance of mechanical output across successive bouts.

Fatigue during repeated-sprint exercise develops rapidly and is influenced by both peripheral and central mechanisms. Peripheral factors include phosphocreatine depletion, the accumulation of metabolic by-products, impaired calcium handling, and changes in muscle excitation–contraction coupling, whereas central factors may involve reduced motor drive and impaired muscle activation [1,4]. The relative contribution of these mechanisms may vary according to sprint duration, the number of repetitions, and recovery interval, which may partly explain protocol-specific responses to beetroot juice (BRJ) supplementation. In addition, experimental evidence has demonstrated a progressive decline in glycolytic energy contribution across repeated maximal sprint bouts, highlighting the importance of metabolic regulation during high-intensity intermittent exercise [5]. These physiological demands have stimulated interest in nutritional strategies that may support muscle function, recovery kinetics, and fatigue resistance during sprint-based exercise.

BRJ is a commonly used dietary nitrate (NO_3_^−^) source that has gained attention as a potential ergogenic aid in athletic populations [6,7]. Following ingestion, dietary NO_3_^−^ is reduced to nitrite (NO_2_^−^) by oral commensal bacteria and can subsequently be converted to nitric oxide (NO), particularly under conditions of low oxygen availability and reduced pH. This NO_3_^−^–NO_2_^−^–NO pathway provides an oxygen-independent mechanism for NO production, which may be particularly relevant during high-intensity exercise [8]. Following acute nitrate ingestion, plasma nitrite concentrations typically peak approximately 2–3 h later, which provides a physiological rationale for administering BRJ several hours before exercise [9]. NO may support exercise performance through vascular and intramuscular mechanisms, including improved blood flow, oxygen delivery, mitochondrial efficiency, calcium handling, glucose uptake, and muscle contractile function [10,11,12,13].

Previous systematic reviews and meta-analyses have reported potential benefits of dietary NO_3_^−^ or BRJ supplementation across several exercise modalities, including endurance, intermittent high-intensity, and fatigue-related exercise tasks [14,15,16,17,18]. However, evidence regarding sprint and repeated-sprint performance remains inconsistent. Some studies have reported improvements in peak or mean power during short-duration all-out sprint efforts following BRJ supplementation [19,20], whereas others have found no significant benefits in repeated-sprint running or sport-specific protocols [21,22]. Previous reviews generally examined dietary nitrate across broad exercise modalities, often emphasizing endurance or intermittent exercise, without specifically synthesizing single- and repeated-sprint outcomes [15,18,23]. The present review addresses this gap by providing an updated, sprint-focused synthesis that also considers associated fatigue-related, neuromuscular, perceptual, and physiological responses.

Variation in the reported findings may reflect differences in exercise modality, sprint duration, recovery intervals, NO_3_^−^ dose, timing of ingestion, supplementation duration, and participant characteristics. Training status, sport type, baseline NO bioavailability, muscle fiber composition, and aerobic fitness may influence responsiveness to dietary NO_3_^−^ [24,25,26,27]. In addition, most studies in this area have been conducted in male participants, while evidence in female athletes remains limited and less consistent [26,28]. To provide a broader assessment of neuromuscular responses associated with sprint performance and fatigue, countermovement jump and handgrip strength were included as secondary outcomes related to muscle function and fatigue, rather than as direct measures or surrogates of sprint performance.

Accordingly, this systematic review and meta-analysis evaluated the effects of BRJ supplementation on (i) single-sprint performance, (ii) repeated-sprint performance, and (iii) associated fatigue-related neuromuscular, perceptual, and physiological outcomes, and examined sex as a potential study-level moderator. Based on the variability of the existing evidence, it was hypothesized that BRJ supplementation would produce outcome-specific rather than consistent improvements across sprint- and fatigue-related outcomes.

## 2. Materials and Methods

This systematic review and meta-analysis was conducted in accordance with the Preferred Reporting Items for Systematic Reviews and Meta-Analyses (PRISMA) guidelines and followed the PICOS framework [29]. The completed PRISMA 2020 checklist is provided in Supplementary Table S1. The protocol was registered in the Open Science Framework (OSF; registration DOI: 10.17605/OSF.IO/3KRE4). The review question, eligibility criteria, search strategy, and primary outcomes were consistent with the preregistered protocol. Countermovement jump and handgrip strength were added after registration when comparable data became available and are therefore reported as post-registration outcomes.

### 2.1. Search Strategy and Study Selection

A systematic literature search was conducted in PubMed, Scopus, and Web of Science. The search strategy combined terms related to NO_3_^−^ supplementation, sprint-based exercise, and performance or fatigue outcomes. The following core search string was used: ((nitrate OR nitrite OR beetroot) AND (“high intensity” OR sprint OR “repeated sprints”) AND (performance OR fatigue)). In Web of Science, the search was applied to the Topic field; in Scopus, to the article title, abstract, and keywords; and in PubMed, to the Title/Abstract field. The lower date limit of 2014 was prespecified to capture a contemporary 10-year evidence window at the time of the initial search in 2024. The search was updated in June 2026 to identify newly published eligible studies. Complete database-specific search strategies, including Boolean operators and field specifications, are provided in Supplementary Table S2.

### 2.2. Eligibility Criteria and Study Selection

Eligibility criteria were defined according to the PICOS framework (Table 1). The population included healthy, physically active adults aged 18–40 years. The intervention was inorganic NO_3_^−^ or NO_2_^−^ supplementation provided as beetroot juice (BRJ). The comparator was a placebo or control condition, including NO_3_^−^-depleted compounds or foods. Eligible outcomes included exercise performance and fatigue-related outcomes during single- or repeated-sprint exercise. Only randomized controlled trials were included.

Studies were included if they (i) investigated the effects of BRJ supplementation on single-sprint or repeated-sprint performance; (ii) used an experimental design in humans; (iii) included healthy, physically active adults aged 18–40 years, including recreationally active, trained, or competitive participants; (iv) were published in English; and (v) were published between 2014 and June 2026. Studies were excluded if they involved animals, children (<18 years), older adults (>40 years), physically inactive participants, or individuals with pathologies or clinical conditions. Conference proceedings, doctoral theses, dissertations, case studies, reviews, and meta-analyses were also excluded.

Duplicate records were removed using Zotero reference manager software (version 6.0.37; Corporation for Digital Scholarship, Vienna, VA, USA). Two reviewers (MNE and SP) independently screened titles and abstracts using Rayyan (Rayyan Systems Inc., Cambridge, MA, USA; web application, accessed June 2026). Full texts were then assessed for eligibility by the same reviewers. Disagreements between the two reviewers were resolved through discussion and consensus; when consensus could not be reached, a third reviewer (VEF-E) acted as an adjudicator.

### 2.3. Data Extraction

A standardized spreadsheet was prepared to extract data from all studies meeting the eligibility criteria. Data extraction was performed independently and in duplicate by two researchers (MNE and SP). Any disagreements were first resolved through discussion and consensus; when consensus could not be reached, a third reviewer (VEF-E) acted as an adjudicator.

Extracted information included sample size, participant characteristics (sex, age, and training status), supplementation dose and strategy, testing protocol, placebo composition, NO_3_^−^/NO_2_^−^ response when available, and the mean and standard deviation for each outcome. Extracted outcomes comprised direct sprint-related performance variables, such as sprint time, power output, work rate, and distance covered, as well as fatigue-related and associated responses, including post-exercise neuromuscular performance, physiological markers, and perceptual measures. Countermovement jump and handgrip strength were considered neuromuscular outcomes related to muscle function and fatigue, rather than direct sprint-performance measures.

When means and standard deviations required for effect size calculation were unavailable, the corresponding authors of 10 studies were contacted, and usable additional data were obtained for six studies. When the required data could not be obtained, only the affected outcome was excluded from the corresponding meta-analysis, while the remaining available outcomes from the study were retained. Data that could not be included quantitatively were considered in the narrative synthesis where appropriate. None of the outcomes included in the quantitative synthesis were reported exclusively as medians and ranges; therefore, no conversion to means and standard deviations was required.

### 2.4. Methodological Quality Assessment

Methodological quality was assessed using the Physiotherapy Evidence Database (PEDro) scale [30]. The PEDro scale rates studies from 0 to 10, with scores of 0–3 considered poor, 4–5 fair, 6–8 good, and 9–10 excellent. The scale evaluates key methodological domains, including random allocation, concealed allocation, baseline comparability, blinding of participants, intervention providers and assessors, adequate follow-up, intention-to-treat analysis, between-group comparisons, and reporting of point estimates and measures of variability.

Two researchers (MNE and SP) independently assessed the methodological quality of the included studies. Any disagreements were resolved through discussion before reaching the final score for each study.

In addition to the PEDro assessment, the risk of bias in the included randomized trials was evaluated using the Cochrane Risk of Bias 2 (RoB 2) tool [31]. For the 22 crossover trials, the RoB 2 extension for crossover trials was applied. The assessment focused on the effect of assignment to the intervention and included bias arising from the randomization process, period and carryover effects, deviations from intended interventions, missing outcome data, measurement of the outcome, and selection of the reported result. The period and carryover domain was considered not applicable to the parallel-group trial. Domain-level and overall judgments were classified as low risk, some concerns, or high risk. Assessments were performed independently by two reviewers (MNE and SP), and disagreements were resolved through discussion and consensus. The assessments focused on the objective performance outcomes included in the review. When judgments did not differ across outcomes within the same study, a single study-level judgment was presented. Risk-of-bias figures were generated using the robvis R package (version 0.3.0).

### 2.5. Statistical Analysis

Meta-analyses were conducted to quantify the effects of the intervention on perceptual, neuromuscular, performance, and physiological outcomes. For each study, standardized mean differences were calculated as Hedges’ g [32], which applies a correction for small sample bias, together with their corresponding 95% confidence intervals (95% CIs). Separate meta-analyses were performed for each outcome variable. Consequently, each study contributed only one effect size to each pooled analysis, although the same study could contribute to different outcome-specific meta-analyses when reporting multiple outcomes. For the crossover trials, within-subject correlations were not consistently reported by the primary studies. Therefore, effect sizes were calculated using the reported means, standard deviations, and sample sizes for each condition without incorporating the within-subject correlation. Consequently, the sampling variance was likely overestimated, resulting in more conservative confidence intervals. Effect sizes were interpreted according to Cohen’s conventions, whereby values of 0.2, 0.5, and 0.8 were considered small, moderate, and large, respectively [33].

Statistical heterogeneity was assessed using Cochran’s Q statistic and quantified using the *I*^2^ statistic. *I*^2^ values of approximately 25%, 50%, and 75% were interpreted as representing low, moderate, and high heterogeneity, respectively [34]. Statistical significance was set at *p* < 0.05.

Random-effects models were used for all pooled analyses to account for the expected clinical and methodological heterogeneity across studies, regardless of the observed level of statistical heterogeneity. Between-study variance (τ^2^) was estimated using the restricted maximum-likelihood (REML) method. This approach was considered appropriate given the variability in sport modality, exercise protocols, BRJ supplementation dose and duration, participant characteristics, training status, and placebo composition.

Potential outliers were identified through the examination of externally studentized residuals, with absolute values greater than 2 considered indicative of potential outliers. Influential studies were evaluated using Cook’s distance values. Publication bias was assessed through visual inspection of funnel plots and formally evaluated using Begg’s rank correlation test and Egger’s regression test [35,36] when the number of available studies permitted these analyses. To explore potential sources of heterogeneity, moderator analyses were performed using mixed-effects meta-regression models, with participant sex included as a categorical moderator. Moderator analyses were considered exploratory and were interpreted cautiously; therefore, no formal adjustment for multiple comparisons was applied. As no statistically significant moderating effects were identified for any outcome, the complete results of these analyses are provided in Supplementary Table S4, whereas only the pooled analyses are presented in the Results Section.

All statistical analyses and forest plots were generated using Jamovi software (Version 2.7.13; The Jamovi Project, Sydney, Australia) with the MAJOR module (version 1.2.0) for meta-analysis, which interfaces with the metafor R package, using random-effects models with the REML estimator. The results are reported as pooled standardized mean differences (SMDs) with corresponding 95% confidence intervals.

### 2.6. Certainty of Evidence

The certainty of evidence for each pooled outcome was assessed using the Grading of Recommendations Assessment, Development and Evaluation (GRADE) approach [37]. Randomized controlled trial evidence was initially rated at high certainty and was evaluated across five domains: risk of bias, inconsistency, indirectness, imprecision, and publication bias. The certainty of evidence was classified as high, moderate, low, or very low. 

## 3. Results

### 3.1. Study Selection

**Figure 1:**
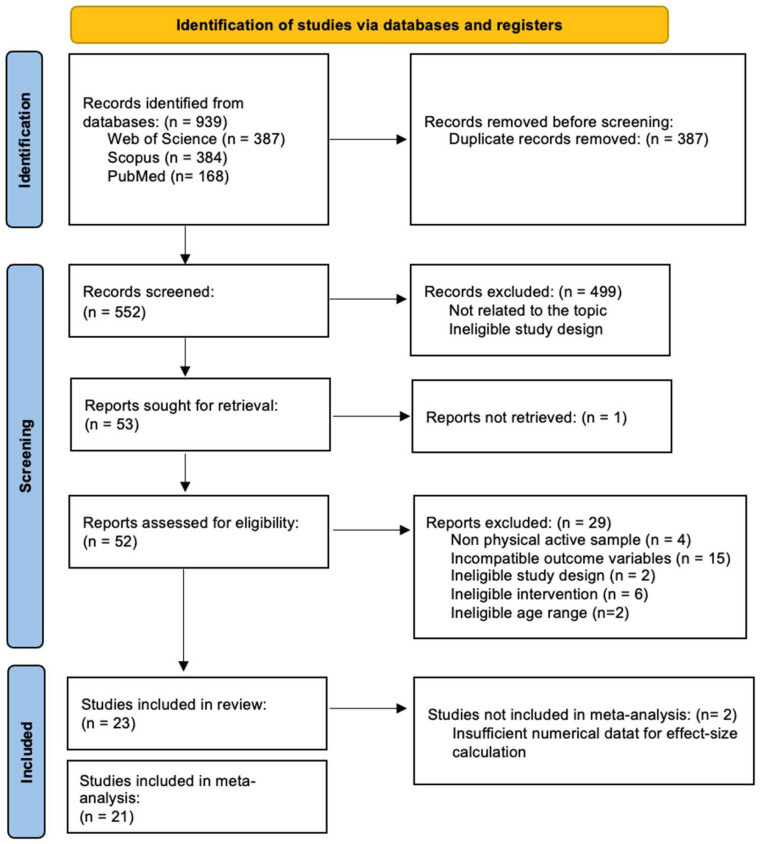
Literature search strategy.

The database search identified 939 records: 387 from Web of Science, 384 from Scopus, and 168 from PubMed. After removing 387 duplicate records, 552 records were screened by title and abstract, of which 499 were excluded. Fifty-three reports were sought for retrieval, and one report could not be retrieved. Fifty-two reports were assessed for eligibility, and twenty-nine were excluded because of non-physically active samples (*n* = 4), incompatible outcome variables (*n* = 15), incompatible study design (*n* = 2), incompatible supplementation (*n* = 6), or incompatible age range (*n* = 2). Finally, 23 studies were included in the systematic review, of which 21 provided sufficient data for meta-analysis. The study selection process is presented in Figure 1.

### 3.2. Study Characteristics

Twenty-two of the twenty-three included studies used randomized crossover designs, whereas Clifford et al. [38] employed a randomized parallel-group design. Most studies were double-blinded, although Pawlak-Chaouch et al. [39] used a single-blind design. Individual study sample sizes ranged from 10 to 52 participants, and the included populations represented a broad range of athletic and physically active groups from different sports and training backgrounds. Placebo composition varied across studies. Most studies used NO_3_^−^-depleted BRJ as the placebo condition (17 of 23 studies). One study used apple–black currant juice [39], two studies used black currant juice [20,40], one used powdered beetroot [41], one used vegetable juice [42] and another used low-fruit squash which was isocaloric and isonitrogenous [38]. In most studies, participant and assessor blinding was achieved by using drink containers that were identical in size, brand, material, and color. Further study details are presented in Table 2.

### 3.3. Methodological Quality and Risk of Bias

The methodological quality of the 23 included studies was assessed using the PEDro scale (Table 3). Total scores ranged from 6 to 10, with a mean score of 8.7, indicating high overall methodological quality. Seven studies [19,20,22,38,42,50,51] achieved a maximum score of 10/10, meeting all assessed criteria. Furthermore, six studies [21,39,41,43,45,46] scored 9 points, classifying them as excellent quality. The remaining studies scored between 7 and 8 [25,28,40,44,47,48,52,53,54], except for Nyakayiru et al. (2017) [49], which had the lowest score of 6/10. Overall, the methodological quality of the included studies was generally good to excellent. However, some criteria, particularly those related to blinding and intention-to-treat analysis, were not consistently met, as detailed in Table 3.

**Figure 2:**
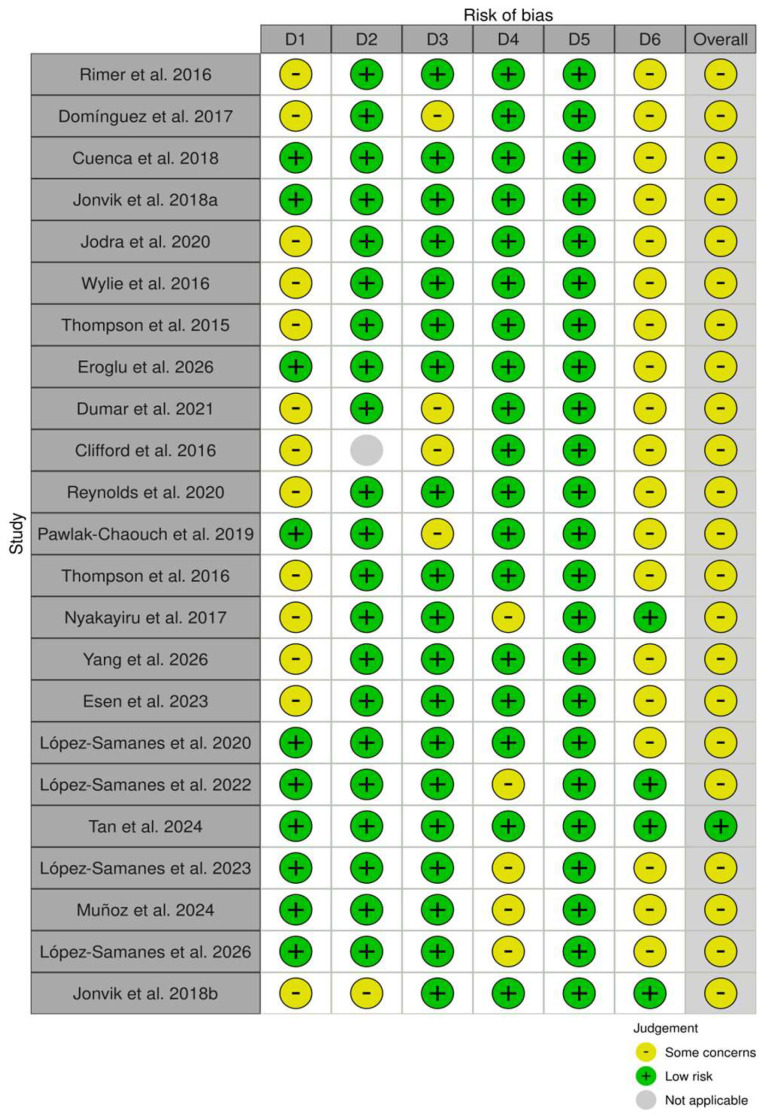
Risk-of-bias assessment of the included randomized trials using the Cochrane RoB 2 tool [19,20,21,22,25,28,38,39,40,41,42,43,44,45,46,47,48,49,50,51,52,53,54].

The RoB 2 assessment identified an overall low risk of bias for one study and some concerns for the remaining 22 studies; no study was judged to be at high risk of bias (Figure 2). Concerns most frequently arose from insufficient reporting of the randomization process and the absence of prospectively published protocols or statistical analysis plans. Specifically, 13 studies presented some concerns in the randomization domain, while 19 studies presented some concerns regarding selection of the reported result. The measurement of outcomes was judged to be at low risk in all studies. The period and carryover domain was considered low risk in 21 crossover studies and raised some concerns in Jonvik et al. [25] because of an observed period effect. This domain was not applicable to Clifford et al. [38], which used a parallel-group design.

D1, bias arising from the randomization process; D2, bias arising from period and carryover effects; D3, bias due to deviations from intended interventions; D4, bias due to missing outcome data; D5, bias in measurement of the outcome; D6, bias in selection of the reported result. The additional crossover domain was incorporated into the overall risk-of-bias judgment. This domain was not applicable to Clifford et al. because this study used a parallel-group design. Green indicates low risk, yellow indicates some concerns, and gray indicates not applicable.

### 3.4. Participant Characteristics

A total of 401 participants were analyzed across the 23 included studies. Regarding sex distribution, most studies were conducted exclusively on male participants (*n* = 18). Three studies recruited only female athletes [22,28,52], while two studies involved mixed cohorts of both males and females [43,44]. Consequently, the total sample comprised of approximately 84% males (*n* = 336) and 16% females (*n* = 65).

The reported mean ages ranged from 18.2 ± 0.4 years [20] to 28.8 ± 3.7 years [28], indicating a predominant focus on young adult populations. In terms of training status, the samples spanned a wide spectrum from ‘recreational’ to ‘elite/Olympic’ levels. The participant profiles included team sport players (e.g., soccer, rugby, hockey, water polo; *n* = 12 studies), as well as cyclists, tennis players, resistance-trained individuals, swimmers, and sprinters. Notably, Jonvik et al. [44] investigated a particularly diverse cohort within a single protocol, including recreational cyclists, national-talent speed skaters, and Olympic-level track cyclists.

### 3.5. BRJ Supplementation Characteristics

Supplementation strategies were categorized as acute, defined as a single-dose protocol, or chronic, defined as a multi-day protocol. Most studies (*n* = 14) used an acute supplementation protocol, administering a single dose of BRJ on the day of the trial [19,20,21,22,28,40,41,42,43,45,50,51,52,54]. The remaining nine studies implemented a chronic loading phase, ranging from 3 to 7 days [25,38,39,44,46,47,48,49,53]. In chronic protocols, the final dose was generally ingested before the experimental trial to coincide with the expected peak in circulating NO_2_^−^ availability.

In studies designed to assess an acute pre-exercise ergogenic effect, BRJ was generally ingested 2 to 3 h before exercise, corresponding to the expected peak in plasma NO_2_^−^ concentration. Clifford et al. [38] used a distinct recovery-oriented protocol in which supplementation began 30 min after the first repeated-sprint test and continued throughout the 72 h recovery period before the second test. The NO_3_^−^ dose varied across studies, ranging from approximately 5.5 mmol [39,41] to 12.8 mmol [20,25,50]. Thirteen studies measured changes in NO_3_^−^, NO_2_^−^, or total NOx concentrations after supplementation, whereas the remaining studies did not assess these responses (Table 2).

### 3.6. Meta-Analysis

#### 3.6.1. Perceptual Response

**Figure 3:**
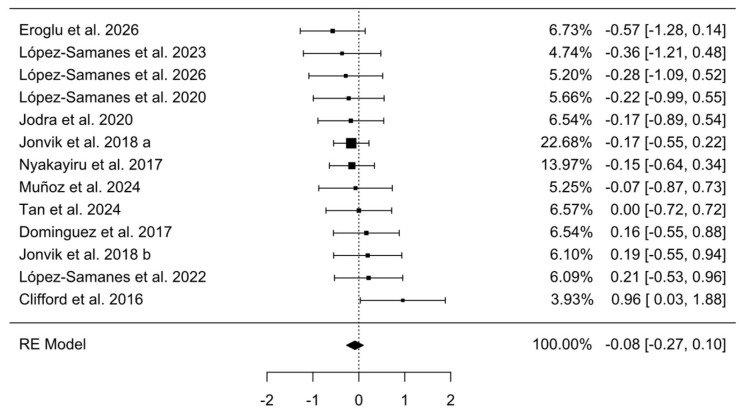
A forest plot of the pooled effect size for the rating of perceived exertion (RPE) [20,22,25,28,38,41,44,45,49,51,52,53,54].

A total of 13 studies were included in the analysis of RPE (Figure 3). BRJ supplementation did not significantly affect RPE (SMD = −0.08, 95% CI: −0.27 to 0.10, *p* = 0.371). No evidence of heterogeneity was observed across studies (τ^2^ = 0.000; *I*^2^ = 0.0%, Q = 9.43, *p* = 0.666).

#### 3.6.2. Neuromuscular Response

**Figure 4:**
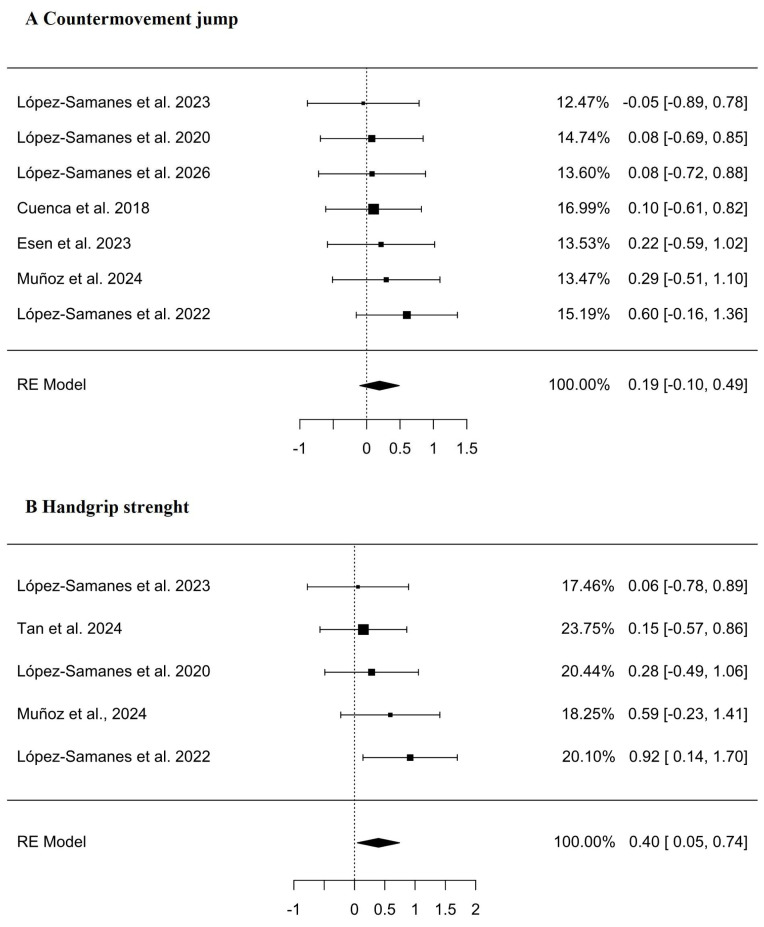
Forest plots of the pooled effect sizes for neuromuscular outcomes [19,22,28,50,51,52,53,54]: (**A**) countermovement jump (CMJ) performance and (**B**) handgrip strength.

Seven studies evaluated CMJ performance (Figure 4A). The pooled effect did not reach statistical significance (SMD = 0.19, 95% CI: −0.10 to 0.49, *p* = 0.199), with no evidence of heterogeneity (τ^2^ = 0; Q(6) = 1.73, *p* = 0.943; *I*^2^ = 0.0%).

Five studies were included in the analysis of handgrip strength (Figure 4B). BRJ supplementation showed a significant small-to-moderate positive effect on handgrip strength (SMD = 0.40, 95% CI: 0.05 to 0.74, *p* = 0.027), with no evidence of heterogeneity (τ^2^ = 0; Q(4) = 3.13, *p* = 0.536; *I*^2^ = 0.0%).

#### 3.6.3. Power-Related Outcomes

**Figure 5:**
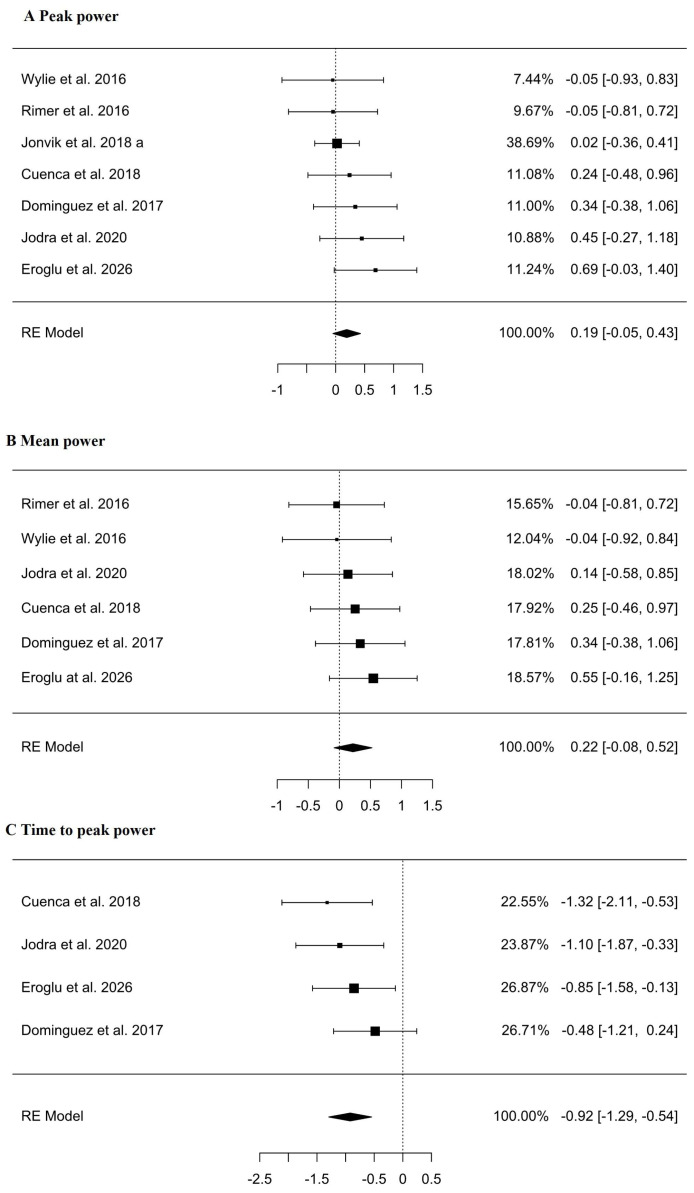
Forest plots of the pooled effect sizes for power-related outcomes [19,20,41,43,44,45,47]: (**A**) peak power, (**B**) mean power, and (**C**) time to peak power.

Seven studies were included in the analysis of peak power (Figure 5A) and six studies in the analysis of mean power (Figure 5B). BRJ supplementation did not significantly affect peak power (SMD = 0.19, 95% CI: −0.05 to 0.43, *p* = 0.118) or mean power (SMD = 0.22, 95% CI: −0.08 to 0.52, *p* = 0.156). No evidence of heterogeneity was observed for either outcome (peak power: τ^2^ = 0; Q(6) = 3.93, *p* = 0.687, *I*^2^ = 0.0%; mean power: τ^2^ = 0; Q(5) = 1.78, *p* = 0.879, *I*^2^ = 0.0%).

Four studies were included in the analysis of time to peak power (Figure 5C). BRJ supplementation showed a significant positive effect on this outcome (SMD = −0.92, 95% CI: −0.54 to −1.29, *p* < 0.001), corresponding to a large effect size. No evidence of heterogeneity was detected (τ^2^ = 0; Q(3) = 2.64, *p* = 0.451; *I*^2^ = 0.0%).

#### 3.6.4. Sprint Performance

**Figure 6:**
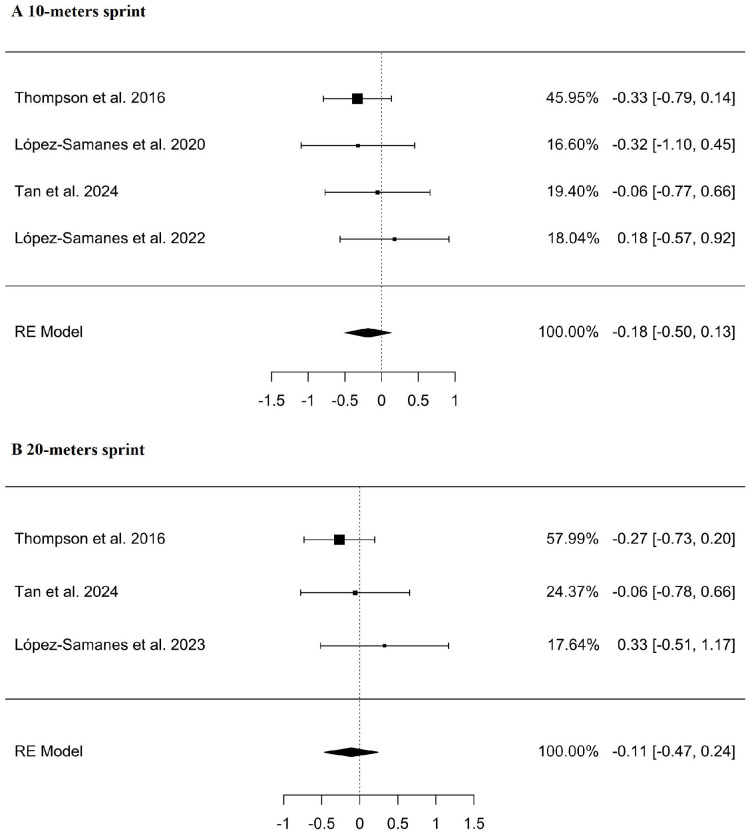
Forest plots of the pooled effect sizes for sprint performance outcomes [22,28,48,51,52]: (**A**) 10 m sprint performance and (**B**) 20 m sprint performance.

Four studies evaluated 10 m sprint performance (Figure 6A). The pooled analysis showed no significant effect of BRJ supplementation on 10 m sprint performance (SMD = −0.18, 95% CI: −0.50 to 0.13, *p* = 0.253). No evidence of heterogeneity was observed across studies (τ^2^ = 0; Q = 1.53, *p* = 0.675; *I*^2^ = 0.0%).

Three studies were included in the analysis of 20 m sprint performance (Figure 6B). BRJ supplementation did not significantly affect 20 m sprint performance (SMD = −0.11, 95% CI: −0.47 to 0.24, *p* = 0.534), and heterogeneity was negligible (τ^2^ = 0; Q = 1.49, *p* = 0.474; *I*^2^ = 0.0%).

#### 3.6.5. Physiological Responses

**Figure 7:**
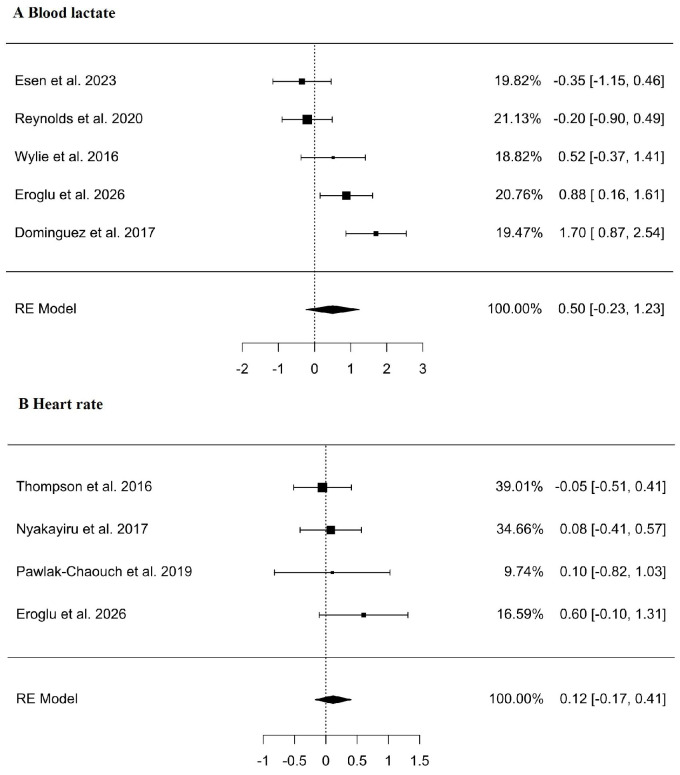
Forest plots of the pooled effect sizes for physiological responses [20,21,39,41,47,48,49,50]: (**A**) blood lactate and (**B**) heart rate.

Five studies were included in the analysis of blood lactate concentration (Figure 7A). BRJ supplementation did not significantly affect blood lactate (SMD = 0.50, 95% CI: −0.23 to 1.23, *p* = 0.180). However, substantial heterogeneity was observed for this outcome (τ^2^ = 0.535; *I*^2^ = 76.98%, Q = 17.20, *p* = 0.002).

Four studies assessed heart rate responses following BRJ supplementation (Figure 7B). The pooled analysis revealed no significant effect on heart rate (SMD = 0.12, 95% CI: −0.17 to 0.41, *p* = 0.449), with no evidence of heterogeneity between studies (τ^2^ = 0.000; *I*^2^ = 0.0%; Q = 0.70, *p* = 0.872).

Outlier and influence diagnostics showed that no study exceeded the threshold for studentized residuals in any of the pooled analyses, indicating the absence of statistical outliers. Similarly, Cook’s distance did not identify influential studies for any outcome except the CMJ analysis, in which one study [52] was identified as potentially influential. No methodological concerns or data inconsistencies were identified that justified excluding this study; therefore, all studies were retained in the primary analyses.

#### 3.6.6. Publication Bias

Publication bias was assessed using funnel plots together with Begg’s rank correlation test and Egger’s regression test whenever at least ten studies were available. Funnel plots are presented in Supplementary Figure S1, and the results of Begg’s and Egger’s tests are summarized in Supplementary Table S3. No statistically significant evidence of publication bias was detected for any outcome evaluated with these methods. However, these findings should be interpreted with caution because several meta-analyses included fewer than ten studies, limiting the power of these tests.

#### 3.6.7. Moderator Analysis

Moderator analyses were conducted to examine the potential influence of sex on the pooled effects. No statistically significant moderating effect of sex was observed for any eligible outcome. However, these findings should be interpreted cautiously because data from female participants were limited. The complete results of these analyses are presented in Supplementary Table S4.

#### 3.6.8. Certainty of Evidence

The GRADE assessment indicated low certainty of evidence for RPE, CMJ, handgrip strength, peak power, mean power, time to peak power, 10 m sprint performance, and heart rate. The certainty of evidence was very low for 20 m sprint performance and blood lactate. The complete GRADE assessment and reasons for downgrading are presented in Supplementary Table S5.

## 4. Discussion

This systematic review and meta-analysis provides an updated synthesis of the effects of BRJ supplementation on performance and fatigue during single- and repeated-sprint exercise in healthy, physically active adults, including trained and competitive athletes. In line with the working hypothesis, the findings indicate that BRJ supplementation produces outcome-specific rather than consistent ergogenic effects. Significant improvements were observed for time to peak power and handgrip strength, whereas no significant effects were found for countermovement jump performance, peak power, mean power, 10 m or 20 m sprint performance, perceived exertion, heart rate, or blood lactate. Importantly, the improvement in handgrip strength should be interpreted as an associated neuromuscular response rather than direct evidence of enhanced sprint performance. These findings broadly agree with previous reviews reporting inconsistent or limited effects of dietary nitrate supplementation on sprint-related power and repeated-sprint performance [23,55,56]. However, previous reviews generally focused on repeated-sprint fatigue resistance, HIIT or sprint-interval power outcomes, or 30 s cycling sprint performance. The present review extends this evidence by incorporating newer trials and evaluating a broader range of single- and repeated-sprint modalities together with neuromuscular, perceptual, and physiological outcomes. Therefore, BRJ supplementation should be considered a potentially context-dependent intervention rather than a broadly effective strategy for all sprint-related performance outcomes.

### 4.1. Effects on Sprint and Power-Related Performance

The present meta-analysis showed that BRJ supplementation did not significantly improve peak power or mean power, although both pooled estimates were in a positive direction. However, given the limited number of studies contributing to these analyses, the possibility that the meta-analysis lacked sufficient power to detect a small true effect cannot be excluded. This finding is consistent with previous reviews suggesting that dietary NO_3_^−^ or BRJ supplementation does not consistently enhance power output during high-intensity sprint-based exercise [55,56]. The individual studies also reflect this variability. Improvements in peak power were reported in several Wingate or very short cycling sprint protocols [19,20,41,43,45], whereas no clear changes were observed in repeated Wingate or repeated-sprint cycling models [44,47]. Similarly, mean power improved in some Wingate-based studies [19,20,40], but remained unchanged in others [41,44,45,47]. These findings suggest that BRJ may exert small favorable effects on power production in selected protocols, but the current evidence is insufficient to support a consistent ergogenic effect on peak or mean power.

In contrast, time to peak power was significantly improved, with a large pooled effect size. This outcome may be more sensitive to BRJ-related effects because it reflects the rapid attainment of maximal power rather than the total magnitude of power output. Several studies using 30 s Wingate or repeated Wingate protocols reported a shorter time to reach peak power after BRJ supplementation [19,20,41,45]. From a mechanistic perspective, this may be relevant to the NO_3_^−^–NO_2_^−^–NO pathway, which has been linked to improved muscle contractile function, type II fiber efficiency, and early-phase force development during high-intensity efforts [13,16]. Therefore, the present findings provide preliminary evidence that BRJ may influence the speed of power development more than maximal or average power production. However, because this finding was based on only four studies using similar Wingate-based protocols, it should be interpreted cautiously, regarded as protocol-specific, and may not be generalizable to other sprint modalities or sport-specific settings.

Nevertheless, the improvement in time to peak power did not translate into consistent gains in short-distance running sprint performance. The pooled analyses showed no statistically significant effects on 10 m or 20 m sprint performance. However, the relatively wide confidence intervals indicate that small beneficial effects cannot be excluded, and these findings should therefore be interpreted with caution. Consistent with these pooled results, most sport-specific studies in tennis, rugby, field hockey, handball, female team-sport athletes, and trained sprinters reported no improvement in these outcomes [28,51,52,53,54]. Only Thompson et al. [48] reported an improvement in 20 m sprint performance following chronic BRJ supplementation in competitive male team-sport athletes. This study differed from several studies reporting null findings by using a 5-day supplementation protocol in a relatively large sample of competitive male team-sport athletes and by confirming increased plasma NO_3_^−^/NO_2_^−^ concentrations. Differences in sample size, supplementation strategy, training status, and biomarker verification may partly explain the inconsistent findings, although the contribution of any single factor cannot be determined. This discrepancy may reflect the greater technical and coordinative demands of running sprint performance, which depends not only on rapid force production but also on acceleration mechanics, stride characteristics, and sport-specific movement efficiency. Accordingly, time to peak power may be a more sensitive outcome for detecting BRJ-related effects than peak power, mean power, or short-distance sprint time. Thus, although the pooled effect on time to peak power was statistically large, its practical significance for real-world athletic performance remains uncertain.

### 4.2. Effects on Repeated-Sprint Ability and Fatigue-Related Outcomes

Narrative findings for direct repeated-sprint and intermittent-sprint performance were mixed. Among the 11 studies using repeated-sprint protocols, four (36%) reported an improvement in at least one direct performance outcome [40,44,46,47], whereas seven (64%) reported no improvement in their primary repeated-sprint performance measures [21,25,28,38,39,42,53]. Null findings were mainly observed in running-based, shuttle-running, swimming, and sport-specific repeated-sprint protocols, including sprint time, fastest sprint time, total sprint time, fatigue index, repeated-sprint ability, and the number of completed repetitions. These findings are consistent with previous reviews suggesting that BRJ or dietary NO_3_^−^ supplementation may have limited effects on direct repeated-sprint performance outcomes, particularly when protocols involve very short sprint bouts with brief recovery periods [16,23]. The changing contributions of the phosphagen, glycolytic, and oxidative energy systems across successive sprint repetitions may partly explain why the effects of BRJ vary according to sprint number, recovery duration, and protocol structure [57].

However, BRJ may be more relevant when the exercise task reflects prolonged intermittent capacity rather than isolated repeated-sprint maintenance. Thompson et al. [46] reported greater total work and faster reaction time during the later phase of an intermittent sprint test, while Wylie et al. [47] found benefits only during the shortest repeated-sprint cycling protocol and not during longer sprint formats. Field-based intermittent tests also showed mixed results: Thompson et al. [48] and Nyakayiru et al. [49] reported improvements in Yo-Yo Intermittent Recovery Test performance, whereas Esen et al. [50] and Tan et al. [22] found no improvement in similar intermittent exercise outcomes. This pattern suggests that BRJ may be more relevant for prolonged intermittent or sport-specific fatigue demands than for repeated-sprint ability itself. One possible explanation is that prolonged intermittent exercise relies more on oxidative metabolism and recovery between efforts, which may be supported by nitrate-derived NO. BRJ may also help maintain fatigue resistance or cognitive-motor performance during the later stages of exercise, although further research is needed. Therefore, current evidence does not support a consistent benefit of BRJ supplementation for repeated-sprint performance, although selected intermittent outcomes may be more responsive.

### 4.3. Physiological, Neuromuscular, and Perceptual Responses

The present meta-analysis showed that BRJ supplementation did not significantly reduce perceived exertion. This finding is consistent with most included studies reporting unchanged general RPE after supplementation, even when some performance-related outcomes improved [20,25,38,40,44,49,52]. A possible explanation is that sprint-based tasks are performed at maximal or near-maximal intensity, where effort perception may be less sensitive to NO_3_^−^-related physiological changes. However, perceptual responses were not completely uniform. Jodra et al. reported lower muscular RPE without changes in general RPE, whereas Yang et al. observed higher RPE after BRJ supplementation [42,45]. Therefore, BRJ does not appear to consistently reduce perceived effort during sprint-based exercise, and the response may depend on whether general or local muscular perception is assessed.

Physiological responses were also limited. The pooled analysis showed no significant effect of BRJ supplementation on heart rate, suggesting that BRJ does not consistently alter cardiovascular strain during short-duration sprint-based exercise (Figure 7B). However, heart rate is a non-specific outcome influenced by exercise intensity, hydration status, environmental conditions, and individual variability; therefore, the absence of a significant effect may have limited physiological relevance. Blood lactate was also not significantly affected overall, but the high heterogeneity indicates that metabolic responses may be protocol-dependent. Accordingly, the pooled estimate should be interpreted with caution, as the substantial between-study heterogeneity suggests that the magnitude of the effect may vary according to study characteristics and exercise protocols. Increased lactate was reported in some Wingate and repeated-sprint cycling protocols [20,41], whereas no change was observed in other cycling, shuttle-running, and Yo-Yo Intermittent Recovery Test protocols [21,47,50]. These differences may reflect variation in exercise mode, total work performed, recovery duration, and glycolytic contribution. Thus, lactate responses should not be interpreted as a uniform physiological effect of BRJ.

Neuromuscular outcomes showed a more specific pattern. Although the pooled estimate suggested a small positive effect on CMJ performance, the confidence interval was compatible with both no effect and a small beneficial effect. Therefore, the available evidence remains inconclusive and these findings should be interpreted with caution. CMJ performance reflects the complex interaction of stretch-shortening cycle function, coordination, and sport-specific neuromuscular control, which may contribute to the variability observed across studies. This is consistent with the absence of CMJ improvement in trained male sprinters reported by López-Samanes et al. [54], although Muñoz et al. [53] reported improved CMJ performance in semi-professional handball players. In contrast, handgrip strength showed a statistically significant small-to-moderate improvement, although this finding should be interpreted with caution given the limited number of studies included and the confidence interval narrowly excluding the null effect (Figure 4B). This finding is compatible with the proposed role of the NO_3_^−^–NO_2_^−^–NO pathway in improving muscle contractile function, type II fiber efficiency, calcium handling, and force production during brief maximal efforts [13,19]. However, handgrip strength should be interpreted as a general neuromuscular indicator rather than a direct measure of sprint performance.

Sport-specific and cognitive–motor outcomes were mixed. Yang et al. [42] reported improvements in choice reaction time and change-of-direction completion time, while simple reaction time was unchanged. Muñoz et al. [53] found no improvement in throwing performance or change-of-direction speed, and Jonvik et al. [25] reported improved dynamic apnea in one period without improvement in swimming intermittent sprint performance. These findings support the working hypothesis that BRJ may have parameter-specific effects rather than broad performance benefits. Overall, BRJ supplementation may influence selected neuromuscular or cognitive–motor outcomes, but these effects do not consistently translate into perceptual, physiological, or sport-specific performance improvements.

### 4.4. Factors Influencing the Ergogenic Response to BRJ Supplementation

The variability observed across outcomes may be explained by differences in supplementation strategy, exercise modality, participant characteristics, and NO_3_^−^/NO_2_^−^ bioavailability. As shown in Table 2, the included studies used acute and multi-day protocols, with NO_3_^−^ doses ranging from approximately 5.5 to 12.8 mmol NO_3_^−^. However, positive and null findings were observed after both supplementation strategies, suggesting that duration alone does not determine the ergogenic response. Moreover, several studies reported increased plasma or salivary NO_3_^−^/NO_2_^−^ concentrations without corresponding improvements in performance, indicating that NO_3_^−^ availability is necessary but not sufficient to ensure a measurable performance benefit [13,23,44]. Because few studies contributed to each pooled outcome and nitrate dose was closely related to other protocol characteristics, a reliable dose-based subgroup analysis or meta-regression was not possible. Therefore, no dose–response relationship can be established, and the pooled estimates should be interpreted as average effects across clinically and methodologically heterogeneous protocols.

Exercise modality and outcome specificity also appear important. Laboratory-based cycling tests may be more sensitive to detecting changes in rapid power development, whereas running, swimming, shuttle-based, and sport-specific protocols involve additional technical and coordinative demands. These include acceleration mechanics, change-of-direction ability, movement efficiency, and tactical or sport-specific skills. Therefore, the absence of consistent effects in field-based protocols may reflect the complexity of sport performance rather than a complete lack of physiological action [14,23,56].

Participant characteristics may further influence responsiveness. The included studies involved recreationally trained individuals, team-sport athletes, resistance-trained participants, sprinters, endurance athletes, and Olympic-level cyclists. Differences in training background, muscle fiber profile, baseline NO availability, aerobic capacity, and sport-specific adaptations may partly explain why BRJ effects were not uniform. This is consistent with the broader view that NO_3_^−^ supplementation may be more effective when the physiological demands of the task match its proposed mechanisms, including improved muscle contractile function, oxygen delivery, and phosphocreatine recovery [13,18].

Finally, sex-specific conclusions remain limited. Although sex was not a statistically significant study-level moderator, this finding should be interpreted cautiously because evidence from female participants remains limited [22,25,28,52]. The non-significant moderator result should therefore not be interpreted as evidence of equivalence or absence of sex-specific responses. Future studies should include adequately powered female samples and consider menstrual cycle phase, hormonal contraceptive use, training status, and sport type when evaluating the ergogenic response to BRJ supplementation.

### 4.5. Limitations and Future Considerations

Several limitations should be considered when interpreting these findings. First, some pooled analyses included a small number of studies, particularly handgrip strength and time to peak power (Figure 4B and Figure 5C). The time-to-peak-power result was based on only four Wingate-based studies, limiting its generalizability to other sprint modalities and sport-specific settings.

Second, methodological variability may have influenced the pooled estimates. As shown in Table 2, the included trials differed in exercise tests, supplementation dose, timing, duration, placebo composition, and whether plasma or salivary NO_3_^−^/NO_2_^−^ responses were measured. Thirteen studies measured post-supplementation NO_3_^−^, NO_2_^−^, or total NOx responses, whereas ten did not provide biomarker verification. Therefore, null findings from studies without biomarker assessment cannot clearly distinguish an insufficient biological response from a true absence of an ergogenic effect. Although most studies attempted to maintain blinding using nitrate-depleted BRJ and similar containers, blinding success was rarely formally assessed. In addition, 22 of the 23 studies used crossover designs, but within-subject correlations were not consistently reported. Consequently, the statistical approach may have produced conservative confidence intervals. The substantial heterogeneity observed for blood lactate also suggests that physiological responses may be protocol-dependent. Publication-bias findings should likewise be interpreted cautiously because most analyses included fewer than ten studies.

Third, participant characteristics, training level, and sport type may influence responsiveness to BRJ supplementation. The pooled estimates combined recreationally active, trained, competitive, and elite participants from team and individual sports, who may differ in aerobic fitness, muscle characteristics, baseline NO bioavailability, sport-specific adaptations, exercise demands, recovery patterns, and energy system contributions. Accordingly, the pooled estimates should be interpreted as average effects across heterogeneous populations and sporting contexts rather than as a uniform response. Female athletes were underrepresented, and the non-significant sex moderator result should not be interpreted as evidence of an absence of sex-specific responses.

The GRADE assessment indicated low certainty for most pooled outcomes and very low certainty for 20 m sprint performance and blood lactate. Accordingly, both significant and non-significant findings should be interpreted cautiously.

Future studies should use larger, sex-balanced samples, include sprint modalities beyond Wingate-based tests, verify NO_3_^−^/NO_2_^−^ responses, and assess individual responsiveness. Greater standardization and clearer reporting of nitrate dose, ingestion timing, placebo composition, exercise protocols, outcome definitions, and measurement time points are also needed. Female-specific factors, including menstrual cycle phase, hormonal contraceptive use, habitual dietary nitrate intake, and baseline NO_3_^−^/NO_2_^−^ status, should also be considered.

## 5. Conclusions

This systematic review and meta-analysis suggests that BRJ supplementation has outcome-specific rather than consistent ergogenic effects on sprint-related performance and associated neuromuscular responses in healthy, physically active adults. BRJ was associated with improvements in time to peak power and handgrip strength; however, the time-to-peak-power finding was based on only four Wingate-based studies, and handgrip strength is an associated neuromuscular outcome rather than a direct measure of sprint performance. BRJ did not significantly improve countermovement jump, peak or mean power, 10 m or 20 m sprint performance, perceived exertion, heart rate, or blood lactate. Therefore, the current evidence does not support BRJ supplementation as a routine strategy to enhance short-distance or repeated-sprint performance. Given the low-to-very-low certainty of evidence, these findings should be interpreted cautiously. Further studies using standardized protocols, verified NO_3_^−^/NO_2_^−^ responses, larger sex-balanced samples, and clearer assessments of individual responsiveness are needed.

## Figures and Tables

**Table 1:** PICOS criteria used for study eligibility.

PICOS Item	Eligibility Criteria
Population	Healthy, physically active adults aged 18–40 years, including trained and competitive athletes
Intervention	Beetroot juice supplementation as a source of inorganic NO_3_^−^ or NO_2_^−^
Comparator	Placebo or control condition, including NO_3_^−^-depleted beetroot juice or matched placebo
Outcomes	Exercise performance and fatigue-related outcomes during single- or repeated-sprint exercise, including sprint time, power output, work rate, distance covered, post-exercise neuromuscular performance, physiological markers, and rating of perceived exertion
Study design	Randomized controlled trials

**Table 2:** Study characteristics included in the systematic review.

Study	Participants	Age (Years)	Supplementation	NO_3_^−^/NO_2_^−^ Response	Exercise Protocol	Main Findings
Rimer et al., 2016 [43]	13 trained athletes (11 M, 2 F)	25.9 ± 7.5	11.2 mmol NO_3_^−^	NM	Maximal inertial-load cycling trials (3–4 s) before and after a 30 s isokinetic cycling test	↑ Pmax ↑ RPMopt ↔ 30 s isokinetic cycling performance
Domínguez et al., 2017 [41]	15 trained male athletes	21.4 ± 1.7	5.6 mmol NO_3_^−^	NM	30 s all-out WAnT	↑ Wpeak ↔ Wmean ↓ time-to-Wpeak ↑ lactate
Cuenca et al., 2018 [19]	15 resistance-trained men	22.4 ± 1.6	6.4 mmol NO_3_^−^	NM	30 s all-out WAnT	↑ Wpeak ↑ Wmean ↓ time-to-Wpeak ↔ CMJ
Jonvik et al., 2018a [44]	52 athletes (29 M, 23 F): recreational cyclists, national-level speed skaters, and Olympic-level track cyclists	27.0 ± 6.0	12.8 mmol·day^−1^ NO_3_^−^ for 6 days	↑ plasma NO_3_^−^ ↑ plasma NO_2_^−^	3 × 30 s WAnT with 4-min active recovery	↔ Wpeak ↔ Wmean ↓ time-to-Wpeak ↔ RPE
Jodra et al., 2020 [45]	15 resistance-trained men	23 ± 2	6.4 mmol NO_3_^−^	NM	30 s all-out WAnT	↑ Wpeak ↓ time-to-Wpeak ↔ Wmean ↓ muscular RPE ↔ general RPE
Dumar et al., 2021 [40]	10 national-level male sprinters	20.3 ± 1.9	6.4 mmol NO_3_^−^	NM	3 × 15 s all-out WAnT with 2-min recovery	↑ Wmean ↑ anaerobic capacity ↑ total work ↔ RPE
Eroglu et al., 2026 [20]	16 male football players	18.2 ± 0.4	12.8 mmol NO_3_^−^	NM	30 s all-out WAnT	↑ Wpeak ↓ time-to-Wpeak ↑ Wmean ↔ heart rate ↔ RPE ↑ lactate
Thompson et al., 2015 [46]	16 male team-sport athletes	24 ± 5	12.8 mmol·day^−1^ NO_3_^−^ for 7 days	↑ plasma NO_3_^−^ ↑ plasma NO_2_^−^	IST: two halves of 20 × 6 s all-out cycling sprints with 114 s recovery	↑ total work ↓ reaction time during the second half
Clifford et al., 2016 [38]	20 male team-sport players	BRJ: 23 ± 3; PL: 21 ± 2	286 mg·day^−1^ NO_3_^−^ for 3 days	NM	RST: 20 × 30 m sprints	↔ sprint time ↔ fastest sprint time ↔ RPE
Wylie et al., 2016 [47]	10 male team-sport players	21 ± 1	8.2 mmol·day^−1^ NO_3_^−^ for 5 days	↑ plasma NO_2_^−^	Repeated-sprint cycling protocols: 24 × 6 s sprints with 24 s recovery; 7 × 30 s sprints with 240 s recovery; 6 × 60 s sprints with 60 s recovery	24 × 6 s: ↑ Wmean, ↔ Wpeak 7 × 30 s: ↔ Wmean, ↔ Wpeak; 6 × 60 s: ↔ Wmean ↑ lactate during 24 × 6 s and 7 × 30 s protocols ↔ lactate during 6 × 60 s protocol
Reynolds et al., 2020 [21]	16 male team-sport athletes	20.9 ± 1.8	6 mmol NO_3_^−^	↑ plasma NO_3_^−^	RST: 10 × 40 m all-out shuttle sprints with 30 s recovery	↔ TT ↔ FT ↔ ST ↔ lactate
Pawlak-Chaouch et al., 2019 [39]	11 elite male endurance athletes	21.7 ± 3.7	340 mg·day^−1^ NO_3_^−^ for 3 days	↑ plasma NOx	SIE: 15 s sprints at 170% intensity with 30 s passive recovery	↔ number of repetitions completed ↔ heart rate
Yang et al., 2026 [42]	21 male soccer players	23.7 ± 3.6	6.4 mmol NO_3_^−^	↑ salivary NO_2_^−^	3 sets of 6 × 20 m maximal sprints with 15 s recovery intervals; agility tests performed immediately after each set	↔ sprint time ↔ SRT ↓ CRT ↓ CODS completion time ↑ RPE
Thompson et al., 2016 [48]	36 competitive male team-sport athletes	24.0 ± 4.0	6.4 mmol·day^−1^ NO_3_^−^ for 5 days	↑ plasma NO_3_^−^ ↑ plasma NO_2_^−^	Maximal 20 m sprints followed by YYIR1	↓ 20 m sprint time ↑ YYIR1 distance ↔ heart rate
Nyakayiru et al., 2017 [49]	32 trained male soccer players	23 ± 1	12.8 mmol·day^−1^ NO_3_^−^ for 6 days	↑ plasma NO_3_^−^ ↑ plasma NO_2_^−^	YYIR1	↑ YYIR1 distance ↔ RPE ↔ heart rate
Esen et al., 2023 [50]	12 trained male rugby union players	20 ± 4	12.8 mmol NO_3_^−^	↑ plasma NO_3_^−^ ↑ plasma NO_2_^−^	YYIR1	↔ YYIR1 performance ↔ CMJ ↔ lactate
Tan et al., 2024 [22]	15 female team-sport athletes	20 ± 1	12.0 mmol NO_3_^−^	↑ plasma NO_3_^−^ ↑ plasma NO_2_^−^	Performance test battery including 10 m and 20 m sprints and YYIR1	↔ 10 m sprint performance ↔ 20 m sprint performance ↔ YYIR1 distance
López-Samanes et al., 2020 [51]	13 highly competitive male tennis players	25.4 ± 5.1	6.4 mmol NO_3_^−^	NM	10 m sprint test	↔ 10 m sprint performance ↔ CMJ ↔ handgrip strength
López-Samanes et al., 2022 [52]	14 semi-professional female rugby players	25.0 ± 3.7	12.8 mmol NO_3_^−^	NM	10 m and 30 m sprint tests	↔ 10 m sprint performance ↔ 30 m sprint performance ↔ RPE ↑ CMJ ↔ handgrip strength
López-Samanes et al., 2023 [28]	11 elite female field hockey players	28.8 ± 3.7	6.4 mmol NO_3_^−^	NM	20 m sprint and RSA test	↔ 20 m sprint performance ↔ RSA ↔ CMJ ↔ handgrip strength
Muñoz et al., 2024 [53]	12 semi-professional male handball players	21.5 ± 5.7	6.4 mmol·day^−1^ NO_3_^−^ for 3 days	↑ salivary NO_3_^−^ ↑ salivary NO_2_^−^	Handball-specific neuromuscular test battery	↔ throwing performance; ↔ CODS ↔ repeated-sprint performance ↑ CMJ ↔ handgrip strength
López-Samanes et al., 2026 [54]	12 trained male sprinters	24.3 ± 4.8	6.4 mmol NO_3_^−^	↑ salivary NO_3_^−^ ↑ salivary NO_2_^−^	60 m and 100 m sprint tests	↔ 60 m sprint performance ↔ 100 m sprint performance ↔ CMJ ↔ handgrip strength
Jonvik et al., 2018b [25]	14 trained female water polo players	22 ± 4	12.8 mmol·day^−1^ NO_3_^−^ for 6 days	↑ plasma NO_3_^−^; ↑ plasma NO_2_^−^	Swimming IST: 16 × 15 m sprints arranged as 4 × 4 blocks with 30 s recovery between blocks	↔ IST performance ↑ dynamic apnea in period II ↔ RPE

Abbreviations: BRJ, beetroot juice; PL, placebo; M, male; F, female; NOx, total nitrate and nitrite concentration; NO_3_^−^, nitrate; NO_2_^−^, nitrite; Wpeak, peak power output; Wmean, mean power output; time-to-Wpeak, time to reach peak power output; RPE, rating of perceived exertion; TT, total time; FT, fastest time; ST, slowest time; Pmax, maximal power output; RPMopt, optimal pedaling rate; WAnT, Wingate anaerobic test; IST, intermittent sprint test; RST, repeated-sprint test; RSA, repeated-sprint ability; YYIR1, Yo-Yo Intermittent Recovery Test Level 1; SIE, sprint interval exercise; CMJ, countermovement jump; CODS, change-of-direction speed; CRT, choice response time; SRT, simple response time; NM, not measured; ↑, significantly higher after BRJ compared with placebo; ↓, significantly lower after BRJ compared with placebo; ↔, no significant difference between BRJ and placebo. For time-based performance outcomes, ↓ indicates a shorter completion time and therefore improved performance, whereas ↑ indicates a longer completion time and therefore impaired performance.

**Table 3:** Methodological quality assessment of the included studies according to the PEDro scale.

Study	Items										Total Score
	1	2	3	4	5	6	7	8	9	10	
Rimer et al., 2016 [43]	✓	✓	✓	✓	✓	×	✓	✓	✓	✓	9/10
Domínguez et al., 2017 [41]	✓	✓	✓	✓	✓	×	✓	✓	✓	✓	9/10
Cuenca et al., 2018 [19]	✓	✓	✓	✓	✓	✓	✓	✓	✓	✓	10/10
Jonvik et al., 2018a [44]	✓	×	✓	✓	✓	×	✓	✓	✓	✓	8/10
Jodra et al., 2020 [45]	✓	✓	✓	✓	✓	×	✓	✓	✓	✓	9/10
Dumar et al., 2021 [40]	✓	×	✓	✓	✓	×	✓	✓	✓	✓	8/10
Eroglu et al., 2026 [20]	✓	✓	✓	✓	✓	✓	✓	✓	✓	✓	10/10
Thompson et al., 2015 [46]	✓	×	✓	✓	✓	✓	✓	✓	✓	✓	9/10
Clifford et al., 2016 [38]	✓	✓	✓	✓	✓	✓	✓	✓	✓	✓	10/10
Wylie et al., 2016 [47]	✓	×	✓	✓	✓	×	✓	✓	✓	✓	8/10
Reynolds et al., 2020 [21]	✓	✓	✓	✓	✓	×	✓	✓	✓	✓	9/10
Pawlak-Chaouch et al., 2019 [39]	✓	✓	✓	×	✓	✓	✓	✓	✓	✓	9/10
Yang et al., 2026 [42]	✓	✓	✓	✓	✓	✓	✓	✓	✓	✓	10/10
Thompson et al., 2016 [48]	✓	×	✓	✓	✓	×	✓	✓	✓	✓	8/10
Nyakayiru et al., 2017 [49]	✓	×	✓	✓	✓	×	×	×	✓	✓	6/10
Esen et al., 2023 [50]	✓	✓	✓	✓	✓	✓	✓	✓	✓	✓	10/10
Tan et al., 2024 [22]	✓	✓	✓	✓	✓	✓	✓	✓	✓	✓	10/10
López-Samanes et al., 2020 [51]	✓	✓	✓	✓	✓	✓	✓	✓	✓	✓	10/10
López-Samanes et al., 2022 [52]	✓	✓	✓	✓	✓	×	×	×	✓	✓	7/10
López-Samanes et al., 2023 [28]	✓	✓	✓	✓	✓	×	×	×	✓	✓	7/10
Muñoz et al., 2024 [53]	✓	✓	✓	✓	✓	✓	×	×	✓	✓	8/10
López-Samanes et al., 2026 [54]	✓	✓	✓	✓	✓	✓	×	×	✓	✓	8/10
Jonvik et al., 2018b [25]	✓	×	✓	✓	✓	×	✓	✓	✓	✓	8/10

✓: Criterion met; ×: Criterion not met. The eligibility criterion was not included in the total score. The 10 scored PEDro items were: 1, random allocation; 2, concealed allocation; 3, baseline comparability; 4, blinding of participants; 5, blinding of intervention providers; 6, blinding of outcome assessors; 7, adequate follow-up; 8, intention-to-treat analysis; 9, between-group comparisons; 10, point estimates and measures of variability.

## Data Availability

The original contributions presented in this study are included in the article/Supplementary Material. Further inquiries can be directed to the corresponding author.

## Supplementary Materials

The following supporting information can be downloaded at: https://www.mdpi.com/article/10.3390/nu18152513/s1, Figure S1: Funnel Plots for the pooled outcomes; Table S1: PRISMA 2020 checklist; Table S2: Database-specific search strategies used in the systematic review; Table S3: Assessment of publication bias across all outcomes using Begg’s rank correlation test and Egger’s regression test; Table S4: Moderator analyses according to participant sex; Table S5: GRADE assessment of the certainty of evidence for the effects of beetroot juice supplementation.

## Abbreviations

BRJ	Beetroot juice
CI	Confidence interval
CMJ	Countermovement jump
CODS	Change-of-direction speed
CRT	Choice reaction time
FI	Fatigue index
FT	Fastest time
HR	Heart rate
NO	Nitric oxide
NO_2_^−^	Nitrite
NO_3_^−^	Nitrate
NOx	Total nitrate and nitrite
OSF	Open Science Framework
PEDro	Physiotherapy Evidence Database
PICOS	Population, Intervention, Comparator, Outcomes, and Study design
PL	Placebo
PRISMA	Preferred Reporting Items for Systematic Reviews and Meta-Analyses
RPE	Rating of perceived exertion
RSA	Repeated-sprint ability
RST	Repeated-sprint test
SD	Standard deviation
SMD	Standardized mean difference
SRT	Simple reaction time
ST	Slowest time
TT	Total time
WAnT	Wingate anaerobic test
Wmean	Mean power output
Wpeak	Peak power output
Wmin	Minimum power output
YYIR1	Yo-Yo Intermittent Recovery Test Level 1
