# Supplementary material for: The Effects of Beetroot Juice Supplementation on Performance and Fatigue During Single and Repeated Sprints: A Systematic Review and Meta-Analysis
Source: Nutrients. 2026 Aug 3;18(15):2513. doi: 10.3390/nu18152513 (PMC13467929; doi:10.3390/nu18152513)

Supplementary Table S1. PRISMA 2020 checklist

Section and Topic	Item #	Checklist item	Location where item is reported
**TITLE**			
Title	1	Identify the report as a systematic review.	p. 1, lines 1–4.
**ABSTRACT**			
Abstract	2	See the PRISMA 2020 for Abstracts checklist.	p. 1, lines 15–39.
**INTRODUCTION**			
Rationale	3	Describe the rationale for the review in the context of existing knowledge.	pp. 2–4, lines 41–103.
Objectives	4	Provide an explicit statement of the objective(s) or question(s) the review addresses.	p. 4, lines 98–103.
**METHODS**			
Eligibility criteria	5	Specify the inclusion and exclusion criteria for the review and how studies were grouped for the syntheses.	pp. 4–5, lines 126–170; Table 1.
Information sources	6	Specify all databases, registers, websites, organisations, reference lists and other sources searched or consulted to identify studies. Specify the date when each source was last searched or consulted.	p. 4, lines 114–125.
Search strategy	7	Present the full search strategies for all databases, registers and websites, including any filters and limits used.	p. 4, lines 114–125; Suppl. Table S2.
Selection process	8	Specify the methods used to decide whether a study met the inclusion criteria of the review, including how many reviewers screened each record and each report retrieved, whether they worked independently, and if applicable, details of automation tools used in the process.	p. 5, lines 144–153.
Data collection process	9	Specify the methods used to collect data from reports, including how many reviewers collected data from each report, whether they worked independently, any processes for obtaining or confirming data from study investigators, and if applicable, details of automation tools used in the process.	pp. 5–6, lines 172–190.
Data items	10a	List and define all outcomes for which data were sought. Specify whether all results that were compatible with each outcome domain in each study were sought (e.g. for all measures, time points, analyses), and if not, the methods used to decide which results to collect.	p. 5, lines 177–188.
	10b	List and define all other variables for which data were sought (e.g. participant and intervention characteristics, funding sources). Describe any assumptions made about any missing or unclear information.	pp. 5–6, lines 177–190; Table 2.
Study risk of bias assessment	11	Specify the methods used to assess risk of bias in the included studies, including details of the tool(s) used, how many reviewers assessed each study and whether they worked independently, and if applicable, details of automation tools used in the process.	p. 6, lines 172–194.
Effect measures	12	Specify for each outcome the effect measure(s) (e.g. risk ratio, mean difference) used in the synthesis or presentation of results.	pp. 5–6, lines 196–236.

Section and Topic	Item #	Checklist item	Location where item is reported
Synthesis methods	13a	Describe the processes used to decide which studies were eligible for each synthesis (e.g. tabulating the study intervention characteristics and comparing against the planned groups for each synthesis (item #5)).	pp. 5–6, lines 172–194 and 237–244.
	13b	Describe any methods required to prepare the data for presentation or synthesis, such as handling of missing summary statistics, or data conversions.	pp. 5, lines 163-170
	13c	Describe any methods used to tabulate or visually display results of individual studies and syntheses.	p. 6, lines 232-236
	13d	Describe any methods used to synthesize results and provide a rationale for the choice(s). If meta-analysis was performed, describe the model(s), method(s) to identify the presence and extent of statistical heterogeneity, and software package(s) used.	p. 5-6, lines 195–236.
	13e	Describe any methods used to explore possible causes of heterogeneity among study results (e.g. subgroup analysis, meta-regression).	p. 6, lines 214–219.
	13f	Describe any sensitivity analyses conducted to assess robustness of the synthesized results.	N/A
Reporting bias assessment	14	Describe any methods used to assess risk of bias due to missing results in a synthesis (arising from reporting biases).	p. 6, lines 222–225.
Certainty assessment	15	Describe any methods used to assess certainty (or confidence) in the body of evidence for an outcome.	p. 6, lines 238–244
**RESULTS**			
Study selection	16a	Describe the results of the search and selection process, from the number of records identified in the search to the number of studies included in the review, ideally using a flow diagram.	p. 8, lines 247–257; Fig. 1.
	16b	Cite studies that might appear to meet the inclusion criteria, but which were excluded, and explain why they were excluded.	p. 8, lines 247–257; Fig. 1.
Study characteristics	17	Cite each included study and present its characteristics.	pp. 8–11, lines 259-280; Table 2.
Risk of bias in studies	18	Present assessments of risk of bias for each included study.	pp. 12–13, lines 281-317; Table 3; Fig. 2.
Results of individual studies	19	For all outcomes, present, for each study: (a) summary statistics for each group (where appropriate) and (b) an effect estimate and its precision (e.g. confidence/credible interval), ideally using structured tables or plots.	Table 2, pp. 8–11; Figures 3–7, pp. 14–18.
Results of syntheses	20a	For each synthesis, briefly summarise the characteristics and risk of bias among contributing studies.	pp. 12-13, lines 281–317; Tables 3; Fig. 2.
	20b	Present results of all statistical syntheses conducted. If meta-analysis was done, present for each the summary estimate and its precision (e.g. confidence/credible interval) and measures of statistical heterogeneity. If comparing groups, describe the direction of the effect.	pp. 14–18, lines 348–429; Figures 3–7.

Section and Topic	Item #	Checklist item	Location where item is reported
	20c	Present results of all investigations of possible causes of heterogeneity among study results.	p. 18, lines 419-423; Suppl. Table S4.
	20d	Present results of all sensitivity analyses conducted to assess the robustness of the synthesized results.	N/A
Reporting biases	21	Present assessments of risk of bias due to missing results (arising from reporting biases) for each synthesis assessed.	p. 18, lines 411–417; Suppl. Fig. S1 and Table S3.
Certainty of evidence	22	Present assessments of certainty (or confidence) in the body of evidence for each outcome assessed.	pp. 18, lines 425-429; Suppl. Table S5.
**DISCUSSION**			
Discussion	23a	Provide a general interpretation of the results in the context of other evidence.	pp. 19–23, lines 431–624.
	23b	Discuss any limitations of the evidence included in the review.	pp. 23, lines 625–662.
	23c	Discuss any limitations of the review processes used.	p. 23, lines 649–655.
	23d	Discuss implications of the results for practice, policy, and future research.	p. 23, lines 656–662.
**OTHER INFORMATION**			
Registration and protocol	24a	Provide registration information for the review, including register name and registration number, or state that the review was not registered.	p. 3, lines 105–108.
	24b	Indicate where the review protocol can be accessed, or state that a protocol was not prepared.	p. 3, lines 105–108.
	24c	Describe and explain any amendments to information provided at registration or in the protocol.	p. 3, lines 105–108.
Support	25	Describe sources of financial or non-financial support for the review, and the role of the funders or sponsors in the review.	p. 24, line 686
Competing interests	26	Declare any competing interests of review authors.	p. 24, line 687
Availability of data, code and other materials	27	Report which of the following are publicly available and where they can be found: template data collection forms; data extracted from included studies; data used for all analyses; analytic code; any other materials used in the review.	p. 24, line 690-691

From: Page MJ, McKenzie JE, Bossuyt PM, Boutron I, Hoffmann TC, Mulrow CD, et al. The PRISMA 2020 statement: an updated guideline for reporting systematic reviews. BMJ 2021;372:n71. doi: 10.1136/bmj.n71

Supplementary Table S2. Database-specific search strategies used in the systematic review.

Database	Search field	Complete search strategy
**PubMed**	Title/Abstract	(("nitrate"[Title/Abstract] OR "nitrite"[Title/Abstract] OR "beetroot"[Title/Abstract]) AND ("high intensity"[Title/Abstract] OR "sprint"[Title/Abstract] OR "repeated sprints"[Title/Abstract]) AND ("performance"[Title/Abstract] OR "fatigue"[Title/Abstract]))
**Scopus**	Article title, abstract, and keywords	TITLE-ABS-KEY(("nitrate" OR "nitrite" OR "beetroot") AND ("high intensity" OR "sprint" OR "repeated sprints") AND ("performance" OR "fatigue"))
**Web of Science**	Topic	TS=(("nitrate" OR "nitrite" OR "beetroot") AND ("high intensity" OR "sprint" OR "repeated sprints") AND ("performance" OR "fatigue"))

Supplementary Table S3. Assessment of publication bias across all outcomes using Begg's rank correlation test and Egger's regression test.

Outcome	Begg's test (τ)	p-value	Egger's test (Intercept)	p-value	Evidence of publication bias
RPE	0.154	0.510	0.940	0.347	No
CMJ	−0.143	0.773	−0.334	0.739	No
Handgrip strength	0.000	1.000	0.241	0.810	No
Peak power	−0.048	1.000	0.797	0.425	No
Mean power	−0.467	0.272	−0.937	0.349	No
Time to peak power	−0.667	0.333	−1.440	0.150	No
Sprint 10 m	0.333	0.750	0.766	0.443	No
Sprint 20 m	1.000	0.333	1.154	0.249	No
Blood lactate	0.200	0.817	0.592	0.554	No
Heart rate	0.667	0.333	0.596	0.551	No

Abbreviations: RPE, rating of perceived exertion; CMJ, countermovement jump; τ, Kendall's rank correlation coefficient.

Note: A p-value < 0.05 was considered indicative of potential publication bias. Because most analyses included fewer than 10 studies, these tests had limited statistical power and should be interpreted cautiously.

Supplementary Table S4. Moderator analyses according to participant sex.

**Outcome**	**k**	**β (Estimate)**	**SE**	**Z**	**p**	**τ^2**	**I^2**
Rating of perceived exertion (RPE)	10	0.090	0.228	0.393	0.694	0.000	0.0%
Peak power	7	−0.351	0.244	−1.440	0.150	0.000	0.0%
Mean power	6	−0.313	0.427	−0.734	0.463	0.000	0.0%
Countermovement jump (CMJ)	7	0.157	0.337	0.467	0.640	0.000	0.0%
Sprint time (10 m)	4	0.384	0.332	1.160	0.248	0.000	0.0%
Sprint time (20 m)	3	0.395	0.326	1.210	0.225	0.000	0.0%
Handgrip strength	5	−0.054	0.373	−0.145	0.885	0.006	3.68%

Abbreviations: β, regression coefficient for the moderator (participant sex); SE, standard error; τ^2, between-study variance estimated using the restricted maximum-likelihood (REML) method; I^2, percentage of total variability attributable to between-study heterogeneity; *k*, number of included studies.

Note: Moderator analyses were only performed for outcomes that included studies with both male and female participants. Outcomes for which all included studies involved a single sex were not eligible for moderator analysis.

Supplementary Table S5. GRADE assessment of the certainty of evidence for the effects of beetroot juice supplementation.

Outcome	Studies, participants	SMD (95% CI)	Risk of bias	Inconsistency	Indirectness	Imprecision	Publication bias	Certainty
RPE	13, n = 241	−0.08 (−0.27 to 0.10)	Serious[1]	Not serious	Not serious	Serious[2]	Not suspected	**Low ⊕⊕◯◯**
CMJ	7, n = 89	0.19 (−0.10 to 0.49)	Serious[1]	Not serious	Not serious	Serious[2]	Not suspected	**Low ⊕⊕◯◯**
Handgrip strength	5, n = 65	0.40 (0.05 to 0.74)	Serious[1]	Not serious	Not serious[3]	Serious[4]	Not suspected	**Low ⊕⊕◯◯**
Peak power	7, n = 136	0.19 (−0.05 to 0.43)	Serious[1]	Not serious	Not serious	Serious[2]	Not suspected	**Low ⊕⊕◯◯**
Mean power	6, n = 84	0.22 (−0.08 to 0.52)	Serious[1]	Not serious	Not serious	Serious[2]	Not suspected	**Low ⊕⊕◯◯**
Time to peak power	4, n = 61	−0.92 (−1.29 to −0.54)[5]	Serious[1]	Not serious	Not serious[6]	Not serious	Serious[7]	**Low ⊕⊕◯◯**
10-m sprint time	4, n = 78	−0.18 (−0.50 to 0.13)	Serious[1]	Not serious	Not serious	Serious[2]	Not suspected	**Low ⊕⊕◯◯**

20-m sprint time	3, n = 62	−0.11 (−0.47 to 0.24)	Serious[1]	Not serious	Not serious	Very serious[8]	Not suspected	**Very low ⊕◯◯◯**
Blood lactate	5, n = 69	0.50 (−0.23 to 1.23)	Serious[1]	Serious[9]	Not serious	Very serious[8]	Not suspected	**Very low ⊕◯◯◯**
Heart rate	4, n = 95	0.12 (−0.17 to 0.41)	Serious[1]	Not serious	Not serious	Serious[2]	Not suspected	**Low ⊕⊕◯◯**

[1] Risk of bias: Downgraded one level because most contributing studies had some concerns, mainly regarding randomization reporting and selective reporting. No study was judged at high risk.

[2] Imprecision: Downgraded one level because the confidence interval crossed the null and included a potentially important effect.

[3] Handgrip indirectness: No downgrade was applied; however, handgrip strength is an associated neuromuscular outcome rather than a direct measure of sprint performance.

[4] Handgrip imprecision: Downgraded one level because the confidence interval ranged from a trivial to a moderate-to-large effect.

[5] Direction: Negative values indicate a shorter time to peak power and therefore favour BRJ.

[6] Time-to-peak-power indirectness: No downgrade was applied, although all studies used Wingate-based protocols, limiting generalizability to other sprint modalities.

[7] Publication bias: Downgraded one level because the evidence was based on four small studies and publication bias could not be excluded reliably.

[8] Very serious imprecision: Downgraded two levels because the confidence interval included potentially important effects in opposing directions and the total information size was very small.

[9] Inconsistency: Downgraded one level because blood lactate showed substantial heterogeneity (I^2 = 76.98%, Q = 17.20, p = 0.002).

Supplementary Figure S1. Funnel Plots for the pooled outcomes.

A. RPE

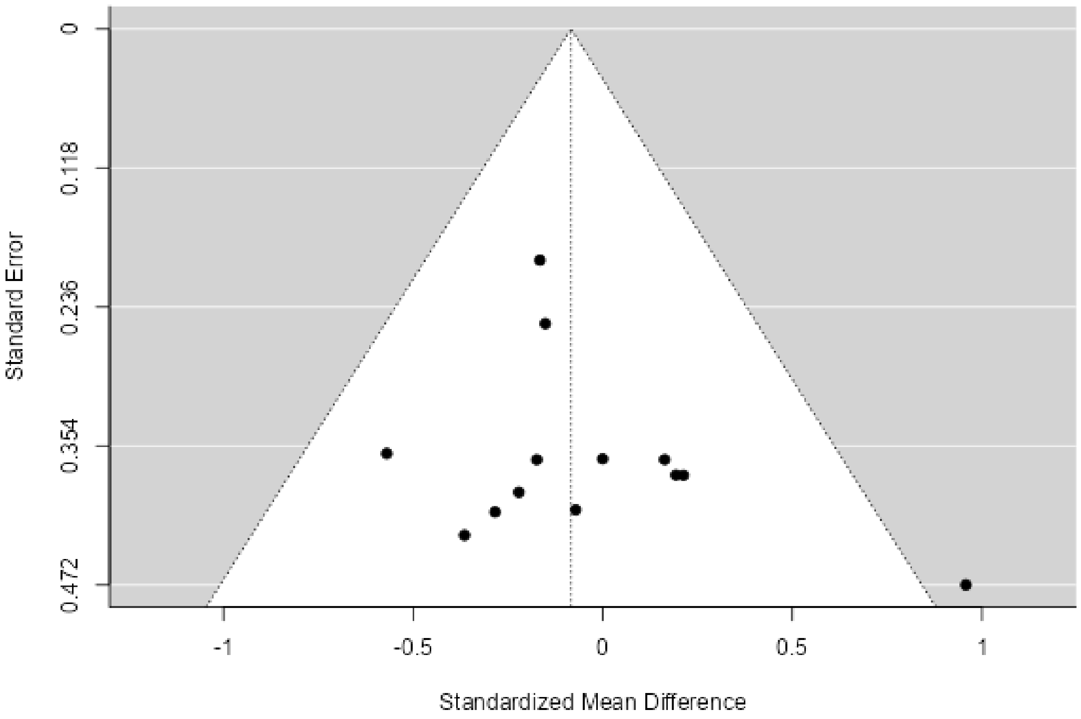

B. CMJ

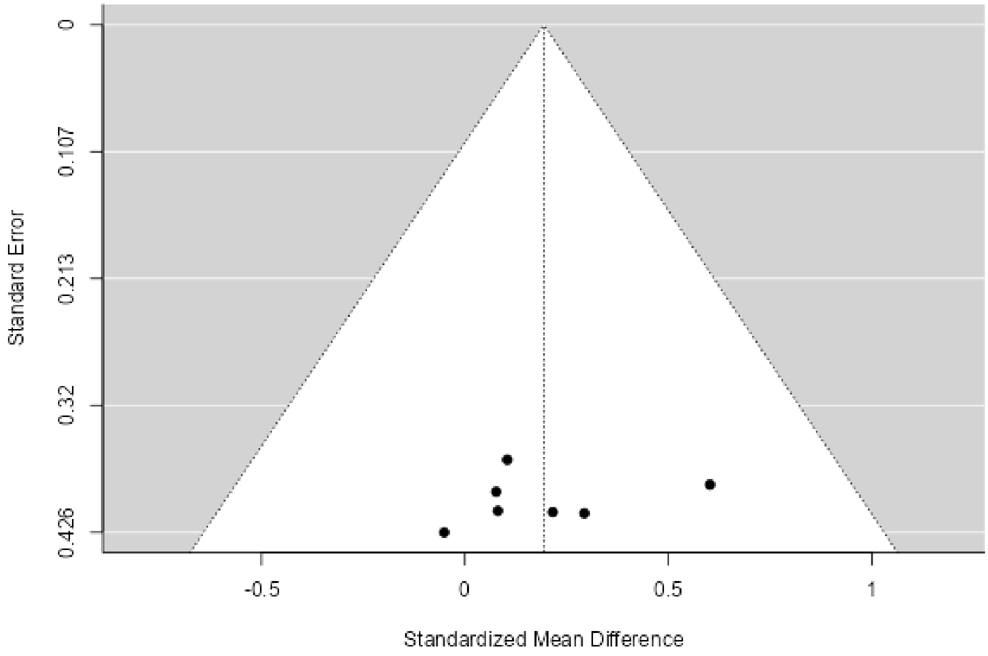

C. Handgrip Strength

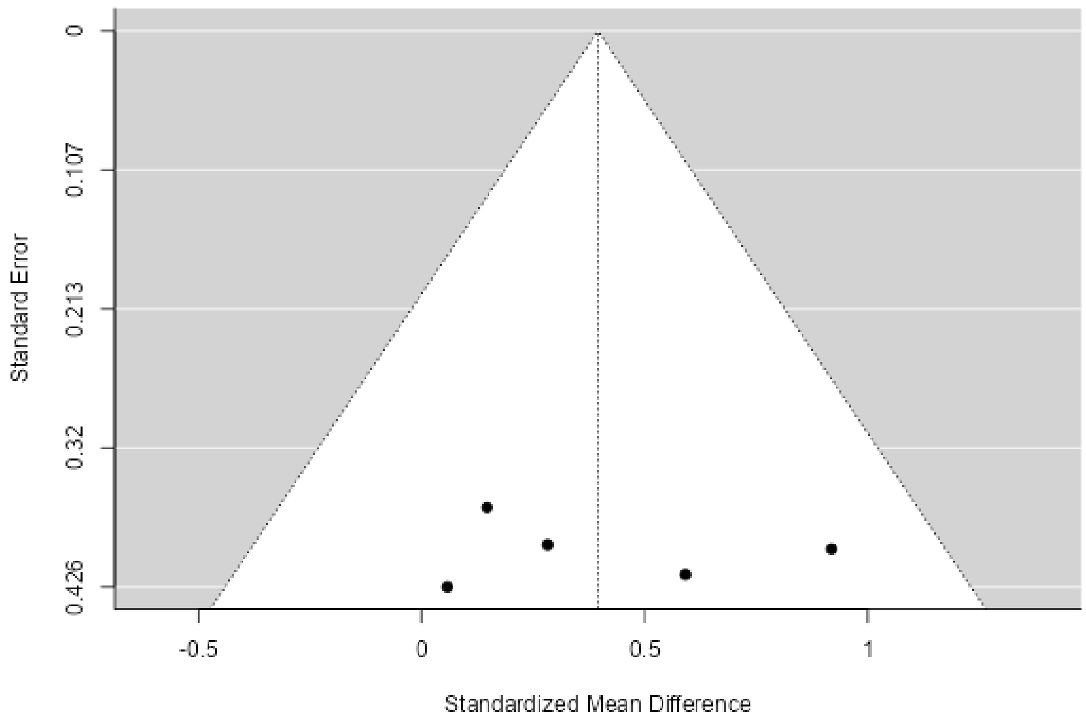

D. Peak Power

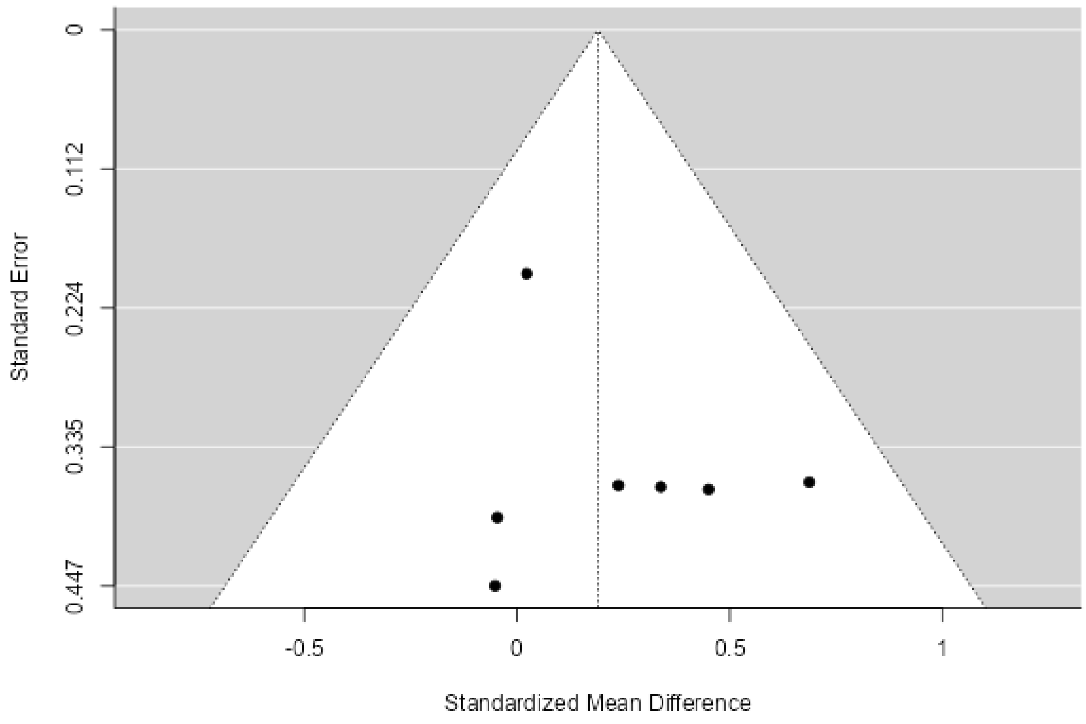

E. Mean Power

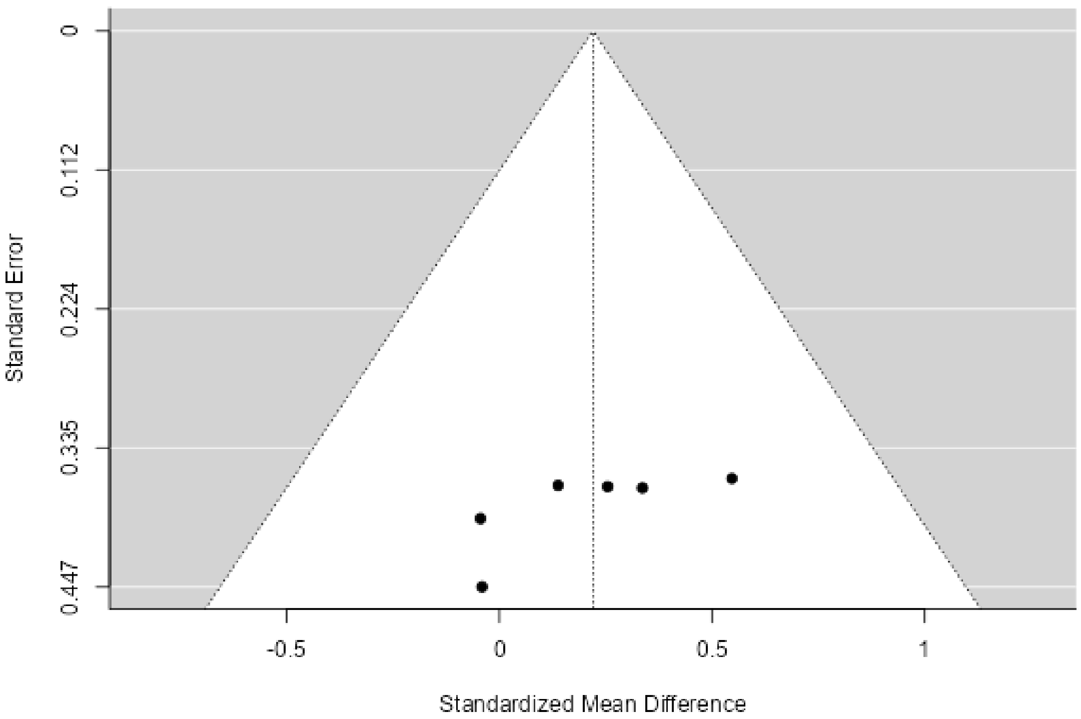

F. Time to Peak Power

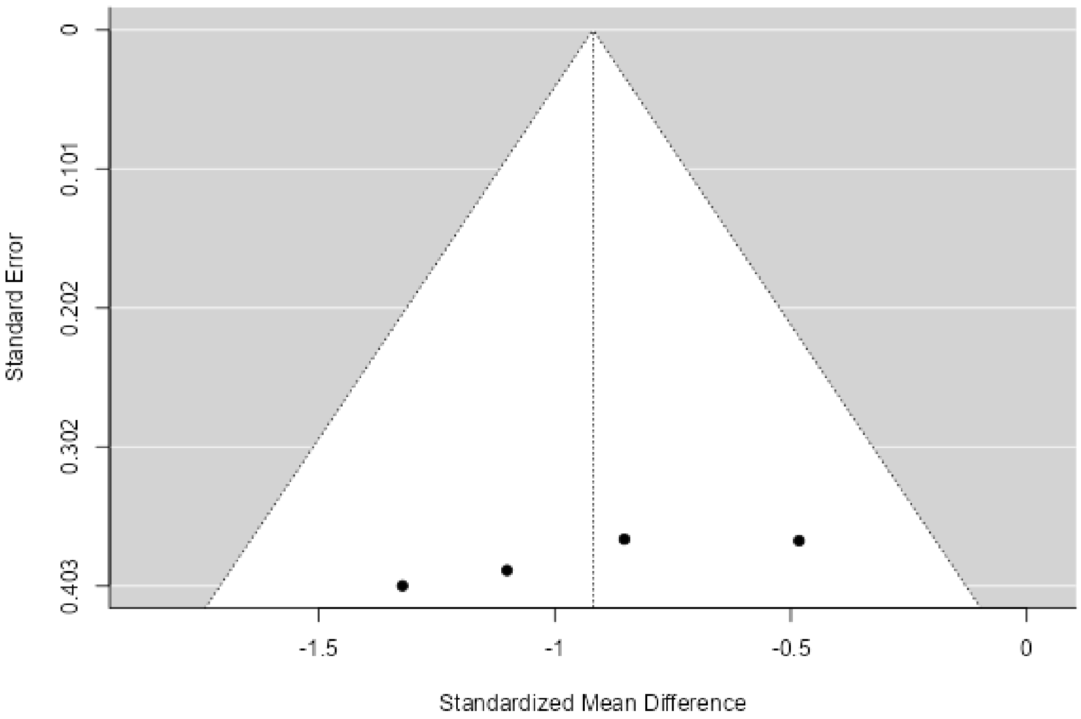

G. 10-m Sprint

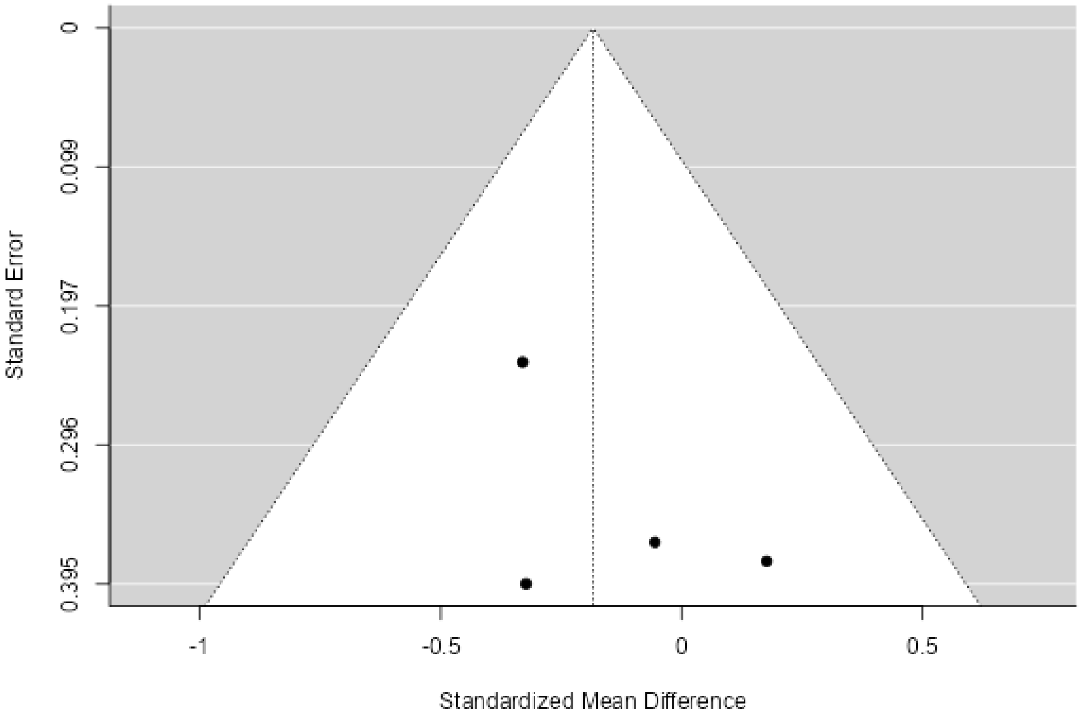

H. 20-m Sprint

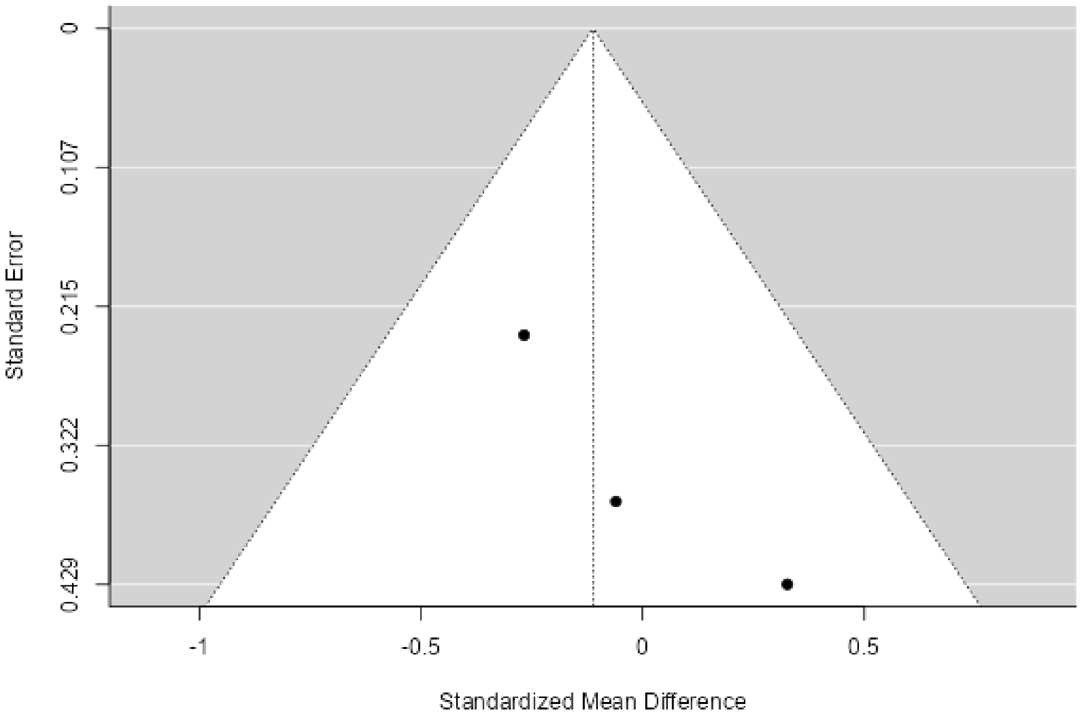

I. Blood Lactate

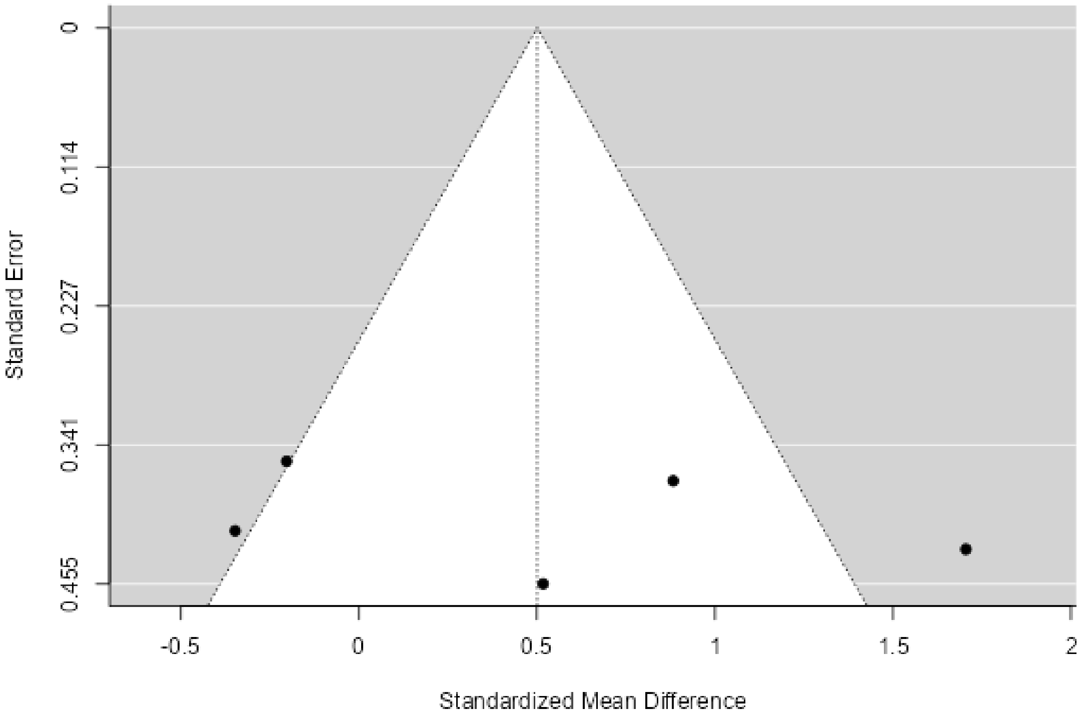

J. Heart Rate

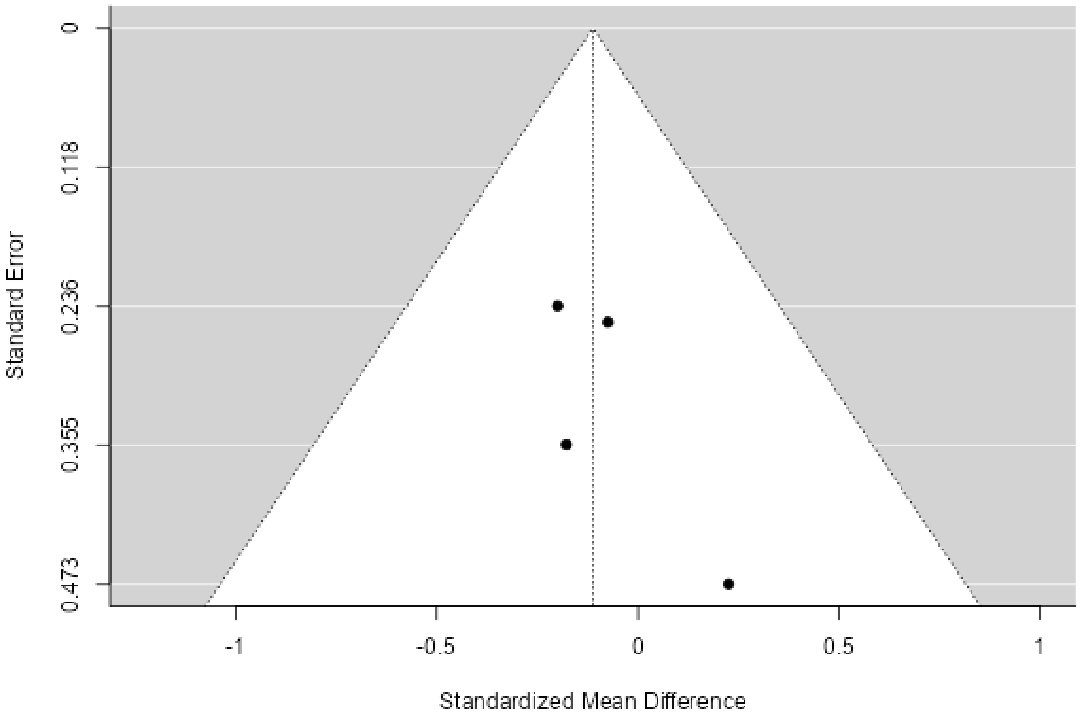

Supplement: Supplementary file 1 [file nutrients-18-02513-s001.zip › nutrients-4431379-supplementary.pdf]
